# Cost-effectiveness of bilateral vs. single internal thoracic artery grafts at 10 years

**DOI:** 10.1093/ehjqcco/qcab004

**Published:** 2021-01-27

**Authors:** Matthew Little, Alastair M Gray, Douglas G Altman, Umberto Benedetto, Marcus Flather, Stephen Gerry, Belinda Lees, Jacqueline Murphy, Mario Gaudino, David P Taggart, for the Arterial Revascularization Trial Investigators

**Affiliations:** Nuffield Department of Population Health, Health Economics Research Centre, University of Oxford, Old Road Campus, Headington, Oxford OX3 7LF, UK; Nuffield Department of Population Health, Health Economics Research Centre, University of Oxford, Old Road Campus, Headington, Oxford OX3 7LF, UK; Nuffield Department of Orthopaedics, Rheumatology & Musculoskeletal Sciences, Centre for Statistics in Medicine, Botnar Research Centre, University of Oxford, Oxford OX3 7LD, UK; School of Clinical Sciences, University of Bristol and Bristol Royal Infirmary, 69 St Michael's Hill, Bristol BS2 8DZ, UK; Norwich Medical School, University of East Anglia and Norfolk and Norwich University Hospital, Norwich Research Park, Norwich, Norfolk NR4 7TJ, UK; Nuffield Department of Orthopaedics, Rheumatology & Musculoskeletal Sciences, Centre for Statistics in Medicine, Botnar Research Centre, University of Oxford, Oxford OX3 7LD, UK; Nuffield Department of Surgical Sciences, University of Oxford, John Radcliffe Hospital, Oxford OX3 9DU, UK; Nuffield Department of Population Health, Health Economics Research Centre, University of Oxford, Old Road Campus, Headington, Oxford OX3 7LF, UK; Department of Cardiothoracic Surgery, Weill Cornell Medicine, New York Presbyterian Hospital, 525 E 68th St, New York, NY 10065, USA; Nuffield Department of Surgical Sciences, University of Oxford, John Radcliffe Hospital, Oxford OX3 9DU, UK

**Keywords:** Healthcare economics, Coronary artery disease, Coronary artery disease surgery, Revascularization, Bypass

## Abstract

**Aims:**

Using bilateral internal thoracic arteries (BITAs) for coronary artery bypass grafting (CABG) has been suggested to improve survival compared to CABG using single internal thoracic arteries (SITAs) for patients with advanced coronary artery disease. We used data from the Arterial Revascularization Trial (ART) to assess long-term cost-effectiveness of BITA grafting compared to SITA grafting from an English health system perspective.

**Methods and results:**

Resource use, healthcare costs, and quality-adjusted life years (QALYs) were assessed across 10 years of follow-up from an intention-to-treat perspective. Missing data were addressed using multiple imputation. Incremental cost-effectiveness ratios were calculated with uncertainty characterized using non-parametric bootstrapping. Results were extrapolated beyond 10 years using Gompertz functions for survival and linear models for total cost and utility. Total mean costs at 10 years of follow-up were £17 594 in the BITA arm and £16 462 in the SITA arm [mean difference £1133 95% confidence interval (CI) £239 to £2026, *P* = 0.015]. Total mean QALYs at 10 years were 6.54 in the BITA arm and 6.57 in the SITA arm (adjusted mean difference −0.01 95% CI −0.2 to 0.1, *P* = 0.883). At 10 years, BITA grafting had a 33% probability of being cost-effective compared to SITA, assuming a cost-effectiveness threshold of £20 000. Lifetime extrapolation increased the probability of BITA being cost-effective to 51%.

**Conclusions:**

BITA grafting has significantly higher costs but similar quality-adjusted survival at 10 years compared to SITA grafting. Extrapolation suggests this could change over lifetime.

## Introduction

The treatment of coronary artery disease places a large economic burden on healthcare systems, with a substantial proportion of that cost arising from coronary artery bypass grafting (CABG).^1^ CABG using a single left internal thoracic artery (SITA) has been found to improve long-term survival and quality of life (QoL) and to be cost-effective in comparison to the alternative of drug-eluting stents-percutaneous coronary intervention (PCI) for patients with severe coronary disease and patients with diabetes in a Dutch context.^2^

The success of SITA grafting has raised interest in the use of bilateral internal thoracic arteries (BITAs) grafting. A recent meta-analysis of 29 observational studies comparing BITA and SITA found BITA was associated with significantly improved long-term survival (hazard ratio 0.78).^3^ However, no previous study has reported a comparison of quality of life, resource use and costs between SITA and BITA.

The Arterial Revascularization Trial (ART) was the first large randomized controlled trial to compare BITA grafting with SITA grafting and was designed with an integrated economic evaluation. Clinical outcomes of ART have recently been published, reporting no significant difference between the two groups for the primary outcome of death from any cause at 10 years of follow-up.^4^ Interim analyses of costs from index admission to 1 year of follow-up and 5 years of follow-up have been published previously,^5^^,^^6^ finding that BITA grafting was associated with 9% higher costs after 1 year, primarily due to longer time in theatre and hospital stay and higher costs associated with sternal wound problems. No further cost differences were found from years 2 to 5, with healthcare costs increasing by approximately £700 per annum in each trial arm. An interim analysis of quality of life scores at 5 years found no significant differences between trial arms in the EQ-5D-3L, SF-36, or Shortened WHO Rose Angina Questionnaire.^7^

The current report presents a cost-effectiveness analysis comparing BITA grafting with SITA grafting for the 3102 patients in ART. Comparison of the two treatments is made in quality-adjusted survival, resource use, and associated costs across 10 years of follow-up. This is the first study to report a randomized comparison of costs and QoL between SITA and BITA grafting.

## Methods

ART randomized patients from 28 centres across seven countries between 2004 and 2007, allocating patients to either SITA or BITA. The trial complied with the Declaration of Helsinki. Ethics approval for UK centres was obtained from the Multi-Centre Research Ethical Committee (MREC), reference number 04/3/006. Prior ethics approval was obtained at each non-UK participating centre and every patient was required to provide written informed consent. Patients were eligible for the trial if they had multi-vessel coronary artery disease and were scheduled to undergo CABG as part of their routine care plan (this included patients requiring urgent surgery, but not those with evolving myocardial infarction). Patients requiring only single grafts or concomitant valve surgery, as well as those with a history of CABG, were excluded. Full details of the trial design including inclusion and exclusion criteria and sample size calculations can be found in the trial protocol.^8^

### Quality of life

Quality of life data were collected at baseline and each follow-up time point using the EuroQol EQ-5D-3L^9^ and the shortened World Health Organization Rose angina questionnaire.^10^ At baseline, 5 years and 10 years the Medical Outcomes Study 36-Item Short-Form Health Survey version 2 (SF-36) was also administered.^11^ The primary analysis makes use of responses from the EQ-5D-3L. EQ-5D-3L values were calculated using the UK population tariff with a score of 1 indicating ‘full health’, 0 death and negative values states worse than death.^12^ Quality-adjusted life years (QALYs) for each patient were derived by combining survival and quality of life data and then calculating area under the curve after linear interpolation between time points.

### Resource use and costs

The perspective of the cost analysis was the English healthcare system, and other costs were not systematically collected. We follow the methods used in several other international trials,^13^^,^^14^ by applying a common set of unit costs to all patients, hence results are reported in pounds sterling using 2017–2018 prices, adjusted where necessary by the GDP deflator index. This avoids the multiple difficulties of collecting sets of unit costs from many countries and then attempting to standardise them using aggregate measures of purchasing power parity.^15^ Mean total costs were derived using the cost of the initial hospital admission combined with annual costs of healthcare contacts and medications across 10 years of follow-up. The costing methodology followed that used in the analyses to 1 year and 5 years of follow-up.^5^^,^^6^ This methodology assigned detailed costs to the initial hospital admission, including the total cost of surgery, post-operative costs, and any in-hospital adverse events (myocardial infarction, cerebrovascular accident, further CABG, further PCI, revascularization with catheter, major bleed, and the cost of hospital stay associated with other adverse events and death). Unit costs were obtained from NHS reference costs where available or from the finance department of one participating UK hospital. Supplementary material online, *Table S1* documents all unit costs and their sources. Reference costs were adjusted for clinical events occurring during the index admission to avoid double counting the cost of stay in hospital.

Resource use data over the 10-year follow-up were collected from case record forms and from a short questionnaire at annual follow-up. Data were collected on numbers of general practitioner (GP) and practice nurse visits, outpatient clinic attendances, cardiac rehabilitation attendance, hospital admission bed days, medication usage, and resource use associated with severe adverse events. GP and nurse visits were costed using estimates from the Personal Social Services Research Unit while NHS reference costs provided unit costs for all recorded hospital outpatient clinic, cardiac rehabilitation clinic visits and costs associated with severe adverse events. The cost of adverse events classed as ‘other’ and death were assumed to be captured by costing the length of stay of the associated hospital admission. An emergency department attendance was assumed where participants were admitted for an event but no overnight stay was reported. The cost of hospital bed days was adjusted to avoid double counting those associated with ‘other’ adverse events and death. Individual medication usage was costed using unit costs from the NHS electronic Market Information Tool (eMIT).

### Cost-effectiveness analyses

The primary analysis compared patients as randomized on an intention to treat basis. Mean resource use items and associated costs, and mean total costs (cost of initial hospital admission plus all healthcare contacts and medications across 10 years of follow-up) were compared using two-sample *t*-tests, while Poisson regression models were used to compare non-zero counts of adverse events. Standard errors were adjusted to account for clustering at the hospital level. Differences in mean QALYs were compared using a linear regression model and were adjusted for imbalances in baseline QoL.^16^ Future QALYs and costs were discounted to present values at an annual rate of 3.5%. Incremental cost-effectiveness ratios (ICERs) were then calculated from the mean difference in QALYs and costs. Cost-effectiveness was evaluated assuming a willingness to pay of £20 000 per QALY and at other levels.^17^ The uncertainty surrounding the ICER estimate was characterized using non-parametric bootstrap replications of the mean difference in QALYs and costs.^18^ Bootstrap samples were taken independently within each treatment group and with resampling at the hospital level. These replications were used to plot the cost-effectiveness plane^19^ and to construct cost-effectiveness acceptability curves, which show the likelihood that the intervention is cost-effective as the willingness to pay changes.^20^

Around a quarter of patients assigned to the SITA arm of the trial received an additional radial-artery graft, while 14% of the BITA group actually underwent SITA grafting. Evidence has grown since the trial was designed that radial-artery grafts are associated with better clinical outcomes in comparison to saphenous-vein grafts,^21^ and so a non-randomized comparison of patients receiving multiple arterial grafts (MAGs) or single arterial grafts (SAGs) was also made. These groups were well balanced in terms of baseline characteristics.^4^ Nonetheless, one-to-one propensity score matching was used to adjust total costs for imbalances in baseline covariates.

The cost-effectiveness of BITA compared to SITA arms beyond the 10 years of the trial was estimated using a Markov cohort model. Survival in each arm of the trial was estimated using Gompertz functions. The first year of trial data was excluded as this improved the fit of the functions to the data. Costs and utility were estimated beyond 10 years with linear models adjusting for age using data from the last 5 years of the trial. This allowed for variation in mean costs and mean utility by age. Patients begin the model at a mean age of 74 years in line with trial participants at 10 years of follow-up, and face an annual probability of death as determined by the survival functions. QALYs and costs in the extrapolation were discounted at an annual rate of 3.5%. Probabilistic sensitivity analysis was conducted to characterize the uncertainty around the extrapolation results using 1000 bootstrap samples from each imputed dataset. The output from the sensitivity analysis is presented using cost-effectiveness acceptability curves.

### Missing data

The rate of missing data was low overall but increased over time, with between 3% and 37% of some resource use items missing at different time points, and between 9% and 38% of QoL data. Missing rates of both resource use and EQ-5D-3L data were similar for BITA and SITA groups. Seventy-one patients had missing vital status, and 279 patients had incomplete adverse event data. Where clinical outcome data were missing, it was assumed an event had not occurred. Logit models of missing total costs and utility across 10-year follow-up on baseline variables showed missing data to be associated with baseline hospital and being a smoker or former smoker at baseline.

Imputation was implemented separately by randomized treatment allocation. EQ-5D-3L data were missing at baseline for 159 (5.1%) patients: these were imputed using mean imputation. All other missing values for both resource use and EQ-5D-3L value data were imputed using chained equations and predictive mean matching.^22^ These equations used baseline hospital, age, sex, baseline Canadian Cardiovascular Society (CCS) class, diabetes, smoking status, peripheral arterial disease, and baseline EQ-5D-3L index. The procedure was repeated to produce 50 imputed datasets with Rubin’s Rule used to summarize across imputations.^23^ The non-parametric bootstrap approach used to construct the estimates for the cost-effectiveness plan and CEAC drew 1000 samples for each imputed dataset.^24^

### Sensitivity analyses

The sensitivity of the results to the missing at random assumption was investigated using a pattern mixture model.^25^ Imputed values were adjusted by a multiplicative scale parameter. The included values of the sensitivity parameter varied both imputed costs and QoL to −20% of their original value at 5% point intervals. The different missing not at random scenarios were then compared in terms of the probability of BITA being cost-effective at a willingness to pay of £20 000 per QALY.

### Subgroup analyses

The cost-effectiveness of BITA compared to SITA was estimated for all patients and for pre-specified patient subgroups: diabetic and non-diabetic, age ≥70 years vs. <70 years, on-pump vs. off-pump, prior myocardial infarction (MI) vs. no prior MI, New York Heart Association (NYHA) class I and II vs. NYHA class III and IV, and Canadian Cardiovascular Society (CCS) class 0, I and II vs. CCS class III and IV. Comparison was also made in each of the three countries (UK, Poland, and Australia) which recruited more than 100 patients to the trial. Linear models with interaction terms between subgroup and treatment allocation were used to test for significant differences in treatment between subgroups, with standard errors again being adjusted for clustering at the hospital level.

## Results

*Table 1* shows resource use, the frequency of adverse events and associated mean cost and QALYs at 10 years for the two trial arms. BITA grafting was associated with significantly larger total mean costs at 10 years of follow-up. This was primarily the result of the significantly higher index admission cost in the BITA group. The BITA group also had significantly higher mean costs associated with outpatient clinic visits and sternal wound problems. There were no significant differences in costs associated with any other healthcare contacts, medication usage or adverse events.

**Table 1:** Resource use, costs, quality-adjusted life years, and cost-effectiveness at 10 year follow-up (intention-to-treat analysis)

	Mean resource use/adverse events at year 10			Mean total cost at year 10		
	SITA (*n* = 1554)	BITA (*n* = 1548)	BITA vs. SITA Mean difference (95% CI, *P*-value)	SITA (*n* = 1554)	BITA (*n* = 1548)	BITA vs. SITA Mean difference (95% CI, *P*-value)
Initial surgery						
Index admission				8819	9475	656 (101, 1212; 0.023)
Discharge cost				562	532	−30 (−353, 292; 0.848)
Healthcare contacts						
GP visits	32	31	−0.67 (−2, 1; 0.461)	1424	1405	−19 (−100, 61; 0.627)
Nurse visits	14	14	0.26 (−1, 2; 0.772)	231	234	3 (−19, 25; 0.784)
Outpatient clinic visits	10	11	0.84 (−1, 2; 0.222)	1538	1683	145 (19, 271; 0.026)
Cardiac rehabilitation visits	10	10	−0.58 (−3, 2; 0.659)	779	736	−43 (−316, 230; 0.750)
Number of nights in hospital^a^	2	2	0.42 (−0, 1; 0.196)	716	875	159 (−163, 481; 0.318)
All health care contacts				4689	4934	245 (−103, 594; 0.159)
Medications						
Total medication	35	35	−0.09 (−1, 1; 0.881)	218	222	5 (−6, 16; 0.383)
SAE treatment^b^						
Myocardial infarction	53	52	0.98 (0.7, 1.4; 0.938)	74	67	−7 (−36, 22; 0.633)
Cerebrovascular accident	63	45	0.72 (0.5, 1.1; 0.088)	129	87	−42 (−94, 10; 0.111)
Further CABG	0	3		0	14	14 (−4, 31; 0.127)
Further PCI	157	154	0.98 (0.8, 1.2; 0.892)	302	300	−2 (−76, 73; 0.965)
Revascularization with catheter	82	79	0.97 (0.7, 1.3; 0.832)	100	107	7 (−46, 60; 0.784)
Sternal wound problems	39	72	1.85 (1.3, 2.7; 0.002)	101	276	175 (34, 316; 0.017)
Major bleeding	11	10	0.91 (0.4, 2.1; 0.834)	48	70	22 (−49, 93; 0.537)
Other AEs (cost of hospital stay only)	1506	1749	1.17 (1.1, 1.2; 0.000)	2201	2377	176 (−322, 675; 0.474)
Death (cost of hospital stay only)	314	297	0.95 (0.8, 1.1; 0.522)	405	363	−41 (−262, 179; 0.703)
All adverse event costs				3360	3662	302 (−290, 894; 0.304)
All costs				17 647	18 825	1178 (204, 2152; 0.020)
Discounted total cost				16 462	17 594	1133 (239, 2026; 0.015)
Life years				9.03	9.05	0.02 (−0.15, 0.20; 0.801)
QALYs^c^				6.57	6.54	−0.01 (−0.2, 0.1; 0.883)
Incremental cost-effectiveness ratio:						BITA dominated [more expensive, less effective (non-significant)]
Probability of cost-effectiveness at willingness to pay threshold						
£13 000						29%
£20 000						33%
£30 000						36%

The cost of index admission includes the total cost of surgery [time in theatre (min), duration-related theatre costs and staff, duration-related anaesthetic costs, time on bypass (min), and other surgery costs (consumables, blood products, aprotinin), post-operative costs (ventilation time, intra-aortic balloon pump, inotropic support, renal support therapy, haemofiltration)] and any in hospital adverse events (myocardial infarction, cerebrovascular accident, further CABG, further PCI, revascularization with catheter, major bleed, other AEs (cost of hospital stay only), death (cost of hospital stay only)].

^a^ Number of nights in hospital exclusive of those associated with an ‘other’ adverse event or death.

^b^ SAE treatment is that occurring in the follow-up period only. The cost of SAEs which occurred during the index admission is included in the cost of index admission.

^c^ Estimated differences in QALYs are adjusted for baseline EQ-5D-3L index.

**Figure 1:**
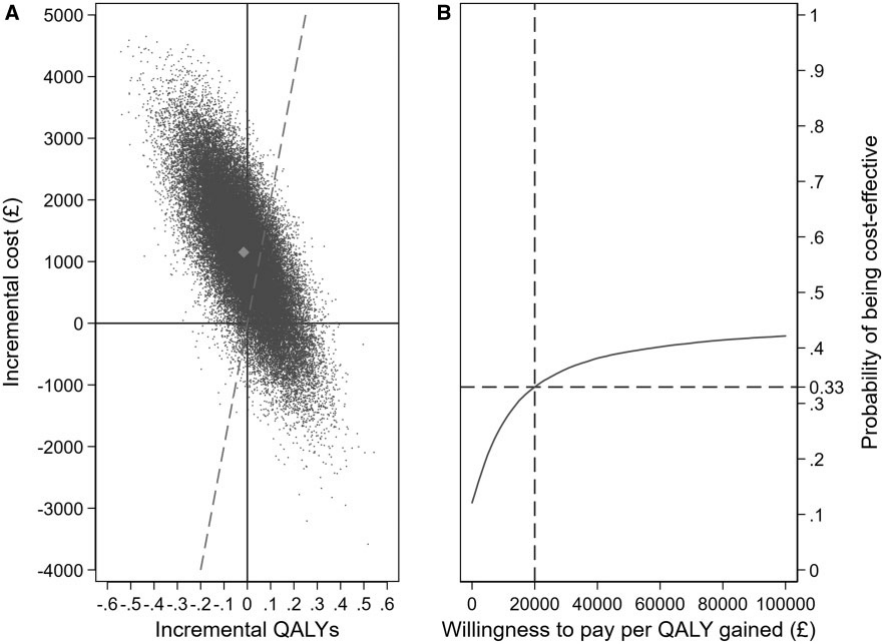
*(A)* Cost-effectiveness plane and (*B*) cost-effectiveness acceptability curve at 10 years of follow-up. (*A*) Plots a series of simulations showing the likelihood that BITA yields more or fewer quality-adjusted life years than SITA (*X*-axis), and costs more or less than SITA (*Y*-axis). Co-ordinates to the left and above the dashed line have a cost-effectiveness ratio above £20 000 per QALY gained; those to the right and below the line are less than £20 000 per QALY gained. (*B*) The probability that BITA is cost-effective compared to SITA as the willingness to pay for each QALY gained is varied from £0 to £100 000, that is, as the dashed line is rotated around the origin.

Mean EQ-5D-3L values initially increased following surgery for both treatment groups but then decreased as patients aged (Supplementary material online, *Tables S2, S3*). No differences were found between the two groups at any time point during the trial (Supplementary material online, *Tables S2, S3*). This was also the case using both the SF-36 and Rose Angina Questionnaire (Supplementary material online, *Tables S4* and *S5*, respectively). By 10 years differences between the two groups in life years and QALYs were small and not statistically significant. Combining the cost [mean difference £1133 95% confidence interval (CI) £239 to £2026, *P* = 0.015] and QALY (adjusted mean difference −0.01 95% CI −0.2 to 0.1, *P* = 0.883) differences, BITA is dominated (more expensive, less effective) than SITA. The probability of BITA grafting being cost-effective compared to SITA (that is, of having a cost per QALY gained of less than £20 000) was 33% (*Figure 1A  *and*  B*).

**Figure 2:**
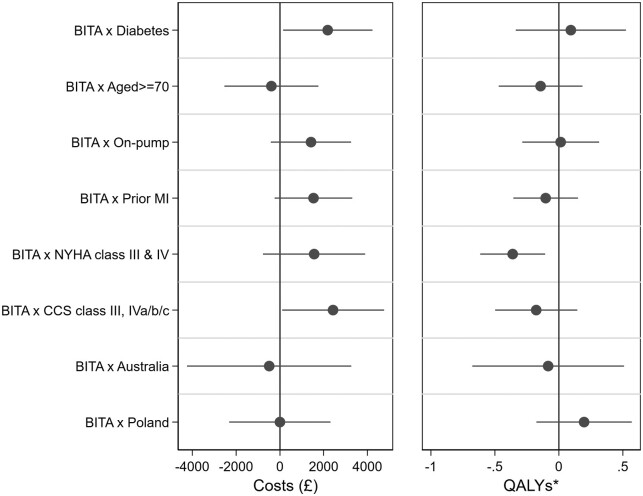
Interaction between treatment allocation and cost or QALY differences by selected subgroups. *Estimated differences in QALYs are adjusted for baseline EQ-5D-3L value.

*Table 2* shows summary results on costs, QALYs and cost-effectiveness for each pre-specified patient subgroup, and *Figure 2* shows these subgroup analyses when tested for interaction between treatment allocation and cost or QALY differences. Concerning costs, a significant interaction with treatment allocation was found for patients with diabetes and for patients with a higher baseline severity CCS class, in both cases the cost difference being larger. A significant interaction was also found between treatment allocation and NYHA class, with classes III and IV being associated with a lower QALY gain: in this group 6.29 QALYs had been accumulated by 10 years in the SITA group compared to 6.01 in the BITA group, a difference of −0.29 (95% CI −0.57, −0.01, *P* = 0.044). Combining these costs and QALY differences, SITA dominated BITA (that is, less costly, more health benefit) in six subgroup analyses. Only in the groups with off-pump surgery, no prior MI, less severe NYHA, and CCS classes and in Poland was the cost of BITA per QALY gained lower than the threshold of £20 000, and in none of these groups was the test for interaction between treatment allocation and either costs or QALYs significant. Further details of these subgroup analyses are provided in Supplementary material online, *Tables S7*–*S13 and Figure S2*.

**Table 2:** Total costs, QALYs, and cost-effectiveness at 10 year follow-up by patient subgroup

	Total cost (£)			QALYs				
	SITA	BITA	BITA vs. SITA Mean difference (95% CI, *P*-value)	SITA	BITA	BITA vs. SITA^a^ Mean difference (95% CI, *P*-value)	ICER	Pr CE
No history of diabetes (*n* = 2368)	16 202	16 803	601 (–453, 1656); 0.251)	6.67	6.61	−0.032 (−0.19, 0.13; 0.688)	Dominated^b^	34%
Diabetes (*n* = 734)	17 315	20 105	2790 (1064, 4516; 0.003)	6.23	6.32	0.061 (−0.35, 0.47; 0.760)	45 642	39%
Aged <70 years (*n* = 2271)	15 276	16 563	1287 (576, 1998; 0.001)	6.76	6.78	0.018 (−0.16, 0.19; 0.836)	72 840	39%
Aged ≥70 years (*n* = 831)	19 601	20 495	894 (–1282, 3070; 0.402)	6.06	5.89	−0.135 (−0.44, 0.17; 0.365)	Dominated^b^	14%
Off-pump (*n* = 1259)	17 204	17 639	435 (–578, 1448; 0.379)	6.51	6.52	0.027 (−0.20, 0.26; 0.805)	13 903	51%
On-pump (*n* = 1819)	16 067	17 781	1714 (297, 3130; 0.020)	6.65	6.64	−0.002 (−0.21, 0.21; 0.986)	Dominated^b^	32%
No prior MI (*n* = 1800)	16 410	16 898	488 (–655, 1631; 0.387)	6.65	6.65	0.026 (−0.18, 0.23; 0.794)	18 903	49%
Prior MI (*n* = 1300)	16 534	18 591	2057 (686, 3428; 0.005)	6.46	6.38	−0.079 (−0.28, 0.12; 0.430)	Dominated^b^	19%
NYHA class I and II (*n* = 2431)	16 451	17 237	786 (–102, 1674; 0.080)	6.64	6.70	0.071 (−0.08, 0.22; 0.338)	11 496	56%
NYHA class III and IV (*n* = 669)	16 494	18 839	2346 (51, 4640; 0.046)	6.29	6.01	−0.290 (−0.57, −0.01; 0.044)	Dominated^b^	15%
CCS class 0, I, II (*n* = 2143)	16 565	16 938	372 (–742, 1486; 0.497)	6.65	6.67	0.046 (−0.13, 0.22; 0.592)	8758	56%
CCS class III, IVa/b/c (*n* = 959)	16 225	19 029	2805 (898, 4711; 0.006)	6.40	6.27	−0.132 (−0.42, 0.16; 0.353)	Dominated^b^	20%
UK (*n* = 2053)	17 173	18 181	(−439, 2476; 0.154) 1008 (–385, 2402; 0.141)	6.40	6.40	0.024 (−0.19, 0.24; 0.810)	42 481	40%
Poland (*n* = 606)	14 615	15 623	(−72, 2084; 0.061) 1007 (–65, 2080; 0.060)	6.79	6.96	0.202 (−0.06, 0.46; 0.091)	4986	65%
Australia (*n* = 192)	18 430	18 948	(−2073, 3240; 0.665) 518 (–2165, 3202; 0.704)	6.59	6.54	−0.058 (−0.65, 0.53; 0.846)	Dominated^b^	39%

^a^ Estimated differences in QALYs are adjusted for baseline EQ-5D-3L index.

^b^ BITA is more expensive but less effective.

Sensitivity analyses in which the assumption that missing data were missing at random was replaced with an assumption that missing cost and QALY data might be systematically different in either arm of the trial, found that the probability of BITA being cost-effective was considerably more sensitive to systematic differences in imputed QALYs than in total costs. For example, a reduction of 10% in the imputed costs in the BITA arm increased the probability of BITA being cost-effective from 33% to 39%. In contrast, a decrease in imputed QALYs of the same magnitude in the SITA arm increased the probability of BITA being cost-effective to 69%. Further results from the sensitivity analysis are shown in Supplementary material online, *Figure S1*.

Results from the non-randomized comparison of multiple vs. SAGs are shown in *Table 3*. MAGs were associated with significantly higher costs in several resource use categories including the index procedure, GP visits, and treatment for sternal wound problems. As a result, total costs were significantly higher over the 10 years of follow-up. However, MAGs were also associated with longer survival over 10 years (9.14 years vs. 8.94, average treatment effect 0.10, CI −0.09, 0.29, *P* = 0.306), and slightly more QALYs were accrued in the MAG group, so that the ICER was £16 412 per QALY gained, with a probability of being cost-effective (less than £20 000) of 54%.

**Table 3:** Resource use, costs, quality-adjusted life years, and cost-effectiveness at 10 year follow-up (non-randomized comparison of multiple vs. single arterial graft)

	Mean resource use/*n* of adverse events at year 10			Mean total cost at year 10		
	SAG (*n* = 1330)	MAG (*n* = 1690)	SAG vs. MAG Mean difference (95% CI, *P*-value)	SAG (*n* = 1330)	MAG (*n* = 1690)	SAG vs. MAG Average treatment effect (95% CI, *P*-value)
Initial surgery						
Index admission				8818	9509	641 (195, 1088; 0.005)
Discharge cost				647	480	−132 (−418, 153; 0.363)
Healthcare contacts						
GP visits	30	33	3 (1, 5; 0.001)	1348	1466	115 (28, 202; 0.010)
Nurse visits	14	13	−1 (−2, 1; 0.521)	239	226	−18 (−47, 12; 0.240)
Outpatient clinic visits	11	11	0 (−1, 2; 0.771)	1597	1632	28 (−198, 254; 0.808)
Cardiac rehabilitation visits	9	10	1 (−1, 4; 0.308)	698	805	98 (−106, 301; 0.347)
Number of nights in hospital^a^	2	2	0.4 (−0, 1; 0.222)	711	870	193 (−92, 478; 0.184)
All healthcare contacts				4593	4998	416 (−38, 870; 0.073)
Medications						
Total medication	35	36	2 (0, 3; 0.017)	218	222	4 (−10, 17; 0.589)
SAE treatment^b^						
Myocardial infarction	38	64	1.33 (0.9, 2.0; 0.169)	62	78	31 (−4, 67; 0.086)
Cerebrovascular accident	55	52	0.74 (0.5, 1.1; 0.126)	122	101	−25 (−86, 37; 0.433)
Further CABG	0	3	.	0	12	14 (−7, 34; 0.181)
Further PCI	141	160	0.89 (0.7, 1.1; 0.327)	315	288	−34 (−140, 72; 0.530)
Revascularization with catheter	75	78	0.82 (0.6, 1.1; 0.215)	106	99	−9 (−60, 43; 0.740)
Sternal wound problems	34	73	1.69 (1.1, 2.5; 0.012)	95	264	45 (−153, 244; 0.656)
Major bleeding	9	11	0.96 (0.4, 2.3; 0.931)	53	65	−9 (−90, 71; 0.824)
Other AEs (cost of hospital stay only)	1283	1871	1.15 (1.1, 1.2; 0.000)	2013	2504	567 (−227, 1360; 0.162)
Death (cost of hospital stay only)	298	293	0.77 (0.7, 0.9; 0.002)	436	352	−58 (−299, 183; 0.637)
All adverse event costs				3201	3763	523 (−458, 1504; 0.296)
All costs				17 478	18 972	1451 (99, 2803; 0.035)
Discounted total cost				16 367	17 681	1251 (63, 2438; 0.039)
Life years				8.94	9.14	0.10 (−0.09, 0.29; 0.306)
QALYs^c^				6.51	6.67	0.08 (−0.1, 0.2; 0.380)
ICER				16 412		
Probability of cost-effectiveness				54%		

^a^ Number of nights in hospital exclusive of those associated with an ‘other’ adverse event or death.

^b^ SAE treatment is that occurring in the follow-up period only. The cost of SAEs which occurred during the index admission is included in the cost of index admission.

^c^ Estimated differences in QALYs are adjusted for baseline EQ-5D-3L index.

*Table 4* and Supplementary material online, *Figure S4* show the results from the extrapolation model. Costs and QoL observed in the trial in each group were extrapolated over an expected lifetime, with competing risks in line with extrapolated survival. The between-group difference in costs remained similar to the 10-year result but the QALY gain associated with BITA grafting showed a small increase, primarily attributable to slightly lower mortality risk in the BITA arm. This resulted in an ICER of £12 962 per QALY and increased the probability of BITA being cost-effective from 33% to 51% at a willingness to pay of £20 000.

**Table 4:** Lifetime cost-effectiveness (extrapolation) of BITA vs. SITA (intention to treat analysis)

	SITA (*n* = 1554)	BITA (*n* = 1548)	BITA vs. SITA
Cost (£)	£21 829	£22 707	£1165
QALYs	12.52	12.61	0.09
Life years	17.65	17.89	0.24
ICER	12 962		
Probability of cost-effectiveness	51%		

## Discussion

This is the first study to report a randomized comparison of costs and quality-adjusted survival between SITA and BITA grafting. We found the higher initial cost of BITA, previously observed at 1 year and at 5 years,^5^^,^^6^ was maintained to 10-year follow-up while no significant differences were observed in QALYs over the same time period. As a result, the probability of BITA being cost-effective at a willingness to pay of £20 000 per QALY gained was only around 33%. There were no significant differences in QoL at any time point across 10 years of follow-up.

### Subgroup analyses

In our analyses of pre-specified subgroups, we found a significant interaction between treatment allocation and difference in costs for patients with diabetes or who were in a higher baseline severity CCS class, both of which were associated with a larger cost difference in favour of SITA. Similarly, we found a significant interaction between treatment allocation and difference in QALYs for patients in NYHA classes III and IV, such that those allocated to SITA accumulated significantly more QALYs over 10 years than those in the BITA group. As a result, we found some evidence that BITA grafting was more cost-effective in groups with less severe baseline conditions and less cost-effective in groups with more severe baseline conditions. Compared to SITA with or without radial artery use, BITA grafting requires sequential (as opposed to simultaneous) harvesting of the conduits and increases the trauma to the chest wall. One hypothesis could therefore be that recovery from the increased surgical trauma is more prolonged and less complete in patients with marginal functional status at baseline, resulting in higher costs and lower quality-adjusted survival compared to SITA in this group.

### Single vs. multiple artery grafts

The non-randomized comparison of patients who received a SAG with those receiving multiple grafts found a survival advantage over 10 years in the MAG group and the resulting cost per QALY gained was well below conventional bounds of willingness to pay for health benefit. These results are suggestive that the use of multiple grafts is preferable to single grafts from an economic perspective as well as from a clinical perspective.

### Longer-term costs and benefits

The costs and benefits of BITA vs. SITA beyond 10 years of follow-up remain an important question. Previously, Buttar *et al.*^3^ conducted a meta-analysis which reviewed both long-term and short-term clinical outcomes following BITA and SITA grafting, and reported overall survival out to more than 20 years after surgery. However, their finding of a significantly lower hazard ratio for the bilateral internal mammary artery group was based on observational studies and is not confirmed by ART at 10 years. Therefore we chose to extrapolate outcomes and costs beyond the end of the trial using a Markov model driven by some fairly simple assumptions on how survival, quality of life and costs would evolve over the remaining lifetime of the trial participants, drawing on ART data. This analysis indicated that the probability of BITA grafting being cost-effective increased to 51%. Continuing follow-up of ART participants beyond 10 years would provide valuable data to test the validity of these assumptions.

### Limitations

The overall level of attrition in the ART trial was exceptionally low,^4^ but the economic analysis, drawing on many different aspects of trial data including questionnaires, case record forms, quality of life and resource use measures and all time points, did face a degree of missing data. We followed best practice in relying primarily on multiple imputation methods to deal with this, and tested the methods using sensitivity analysis. This indicated that the results were robust to large changes in the level of the imputed cost data, but were more sensitive to changes in the values of imputed quality of life data. This is a common result in sensitivity analysis of imputed data in trial-based cost-effectiveness analysis^25^ and highlights the importance of minimizing missing QoL data.

ART was an international study, enrolling patients from seven countries. We conducted this analysis from the perspective of the UK only, applying UK unit costs and quality of life valuations to all observed data. This can be justified on the grounds that the approach has been adopted in a number of other international trial analyses,^13^ two-thirds of all ART patients were recruited in the UK, and the subgroup analyses found no significant interactions between treatment allocation and country for costs or QALYs. However, it remains possible that the results were influenced by variations in treatment pathways between countries.

Although our subgroup analyses were suggestive that BITA grafting was more cost-effective in groups with less severe baseline conditions, it is possible that these results arise from confounding: for example, approximately 22% of patients in NYHA classes I and II had a radial artery graft compared to 17% in classes III and IV. Similarly, the proportion of patients with ejection fraction <50% was 26% in NYHA classes I and II compared with 31% in classes III and IV.

More generally, the results of the intention-to-treat analysis of ART may have been affected by important confounders such as the high crossover rate and the high use of the radial artery in both groups.^26^

Finally, while the results of our as-treated analysis support use of multiple arterial over single grafts from an economic as well as a clinical perspective, we acknowledge that the limitations of non-randomized comparisons may have biased this result. A randomized comparison of multiple arterial and single grafts will be provided by the ROMA trial.^27^

## Conclusions

We found that BITA grafting incurs higher costs than SITA during the initial procedure which are not offset by cost savings in later years. There are no significant differences in quality-adjusted survival at 10 years and so the likelihood that BITA is cost-effective compared to SITA at 10 years is low. However, our extrapolation suggested that BITA may become more cost-effective over a lifetime horizon. Uncertainty surrounding our extrapolation results can best be reduced by continuing to follow-up ART patients for as long as possible.

## Supplementary material

Supplementary material is available at *European Heart Journal – Quality of Care and Clinical Outcomes* online.

qcab004_Supplementary_Data
