# Supplementary material for: Cost-effectiveness of bilateral vs. single internal thoracic artery grafts at 10 years
Source: Eur Heart J Qual Care Clin Outcomes. 2021 Jan 27;8(3):324–32. doi: 10.1093/ehjqcco/qcab004 (PMC9071553; doi:10.1093/ehjqcco/qcab004)
Supplement: qcab004_Supplementary_Data [file qcab004_supplementary_data.docx]

**Centres participating in the Arterial Revascularisation Trial (ART). Principal**

**Investigators (shown in bold), co-investigators and co-ordinators (number of patients enrolled).**

1. John Radcliffe Hospital, Oxford, UK. **D Taggart**, C Ratnatunga, S Westaby, J

Cook, C Wallis (427)

2. Medical University of Silesia (2nd Department of Cardiac Surgery), Katowice,

Poland**. S Wos, M Jasinski, M Deja**, K Widenka, A Blach, R Gocol, D

Hudziak, P Zurek, R Bachowski, R Mrozek, T Kargul, W Domarardzki, J

Frackiewicz (256)

3. Edinburgh Royal Infirmary, Edinburgh, UK. **V Zamvar**, D Ezakadan (217)

4. Austin and Repatriation Medical Centre, Melbourne, Australia. **B Buxton, S**

**Seevanayagam**, G Matalanis, A Rosalion, J Negri, S Moten, V Atkinson, A

Newcomb, P Polidano, R Pana, S Gerbo (192)

5. University Hospital of Wales, Cardiff, UK, **P O’Keefe**, U von Oppell, D Mehta,

A Azzu, A Szafranek, E Kulatilake, J Evans, N Martin, D Banner (185)

6. Royal Sussex County, Brighton, UK. **The late A Forsyth, U Trivedi,** J Hyde,

A Cohen, M Lewis, E Gardner, A MacKenzie, N Cooter, E Joyce, J Parker, F

Champney (180)

7. Freeman Hospital, Newcastle, UK. **S Clark**, J Dark, K Tocewicz, T Pillay, S

Rowling, J Adams-Hall (152)

8. Medical University of Silesia (1st Department of Cardiac Surgery), Katowice,

Poland. **A Bochenek, M Deja**, M Cisowski, M Bolkowski, W Morawski, M

Guc, M Krejca , M Wilczyn-ski, A Duralek, W Gerber, J Skarysz, R Shrestha,

W Swiech, P Szmagala, L Krzych, A Pawlak, K Kepa (145)

9. Royal Infirmary, Manchester, UK. **R Hasan**, D Keenan, B Prendergast, N

Odom, K McLaugh-lin, G Cummings-Fosong, C Mathew, H Iles-Smith, A

Oomen (115)

10. King's College Hospital, London, UK. **J Desai**, A El-Gamel, L John, O

Wendler, M Andrews, K Rance, R Williams, V Hogervorst, J Gregory, J

Jessup, A Knighton, A Hoare (114)

11. Royal Papworth Hospital, Cambridge, UK. **A Ritchie, C Choong, S Nair, C**

**Sudarshan**, D Jenkins, S Large, M Barman, K Dhital, T Routledge, B

Rosengard, H Munday, K Rintoul, E Jarrett, S Lao-Sirieix, A Wilkinson, L

Garner, J Osmond, H Holcombe (101)

12. Castle Hill Hospital, Hull, UK. **A Cale**, S Griffin, J Dickson, J Cook (97)

13. Glenfield Hospital, Leicester, UK. **T Spyt, A Gershlick**, M Hickey, A Sosnowski,

G Peek, J Szostek, L Hadjinikalaou,, E Logtens, M Oakley, S Leji

(95)

14. Harefield Hospital, London, UK. **J Gaer**, M Amrani, G Dreyfus, T Bahrami,

F de Robertis, K Baig, G Asimakopoulos, H Vohra, V Pai, S Tadjkarimi, Soleimani,

G Stavri, G Bull, H Collappen (94)

15. John Paul II, Krakow, Poland. **J Sadowksi, B Kapelak**, B Gaweda, P

Rudzinski, J Stolinski, J Konstanty-Kalandyk, (92)

16. Heart Institute of Pernambuco, Recife, Brazil. **F Moraes**, C Moraes, J Wanderley

(82)

17. Royal Brompton Hospital, London, UK. **J Pepper**, A De Souza, M Petrou, R

Trimlett, T Morgan, J Gavino, SF Wang (82)

18. St George’s Hospital, London, UK. **V Chandrasekaran**, R Kanagasaby, M

Sarsam, H Ryan, L Billings, L Ruddick, A Achampong, E Forster, E Mohama,

P Mc Donnell (78)

19. Medical University of Gdansk, Gdansk, Poland. **R Pawlaczyk**, P Siondalski, J

Rogowski, K Roszak, K Jarmoszewicz, D Jagielak, S Gafka (74)

20. Care Hospital, Hyderabad, India. **G Mannam**, L Rao Sajja, B Raju Dandu, G

Naguboyina, A Yalla, J Peddireddy (69)

21. Northern General Hospital, Sheffield, UK. **N Briffa**, P Braidley, G Cooper, A

Knighton, K Al-len, G Sangha, C Bridge, H McMellon, P Shaw (67)

22. Ospedale Mauriziano, Turin, Italy. **R Casabona, G Actis Dato,** G Bardi, S

Del Ponte, Forsen-nati, F Parisi, G Punta, R Flocco, F Sansone, E Zingarelli,

A Demartino (60)

23. The Cardiothoracic Centre, Liverpool, UK. **W Dihmis, M Kuduvali**, C

Prince, H Rogers, L McQuade, A Duran-Rosas (50)

24. Szpital Uniwersytecki, Bydgoszcz, Poland. **L Anisimowicz**, M Bokszanski, W

Pawliszak, J Kolakowski, G Lau, W Ogorzeja, I Gumanska, P Kulinski (23)

25. Landesklinikum, St Polten and Center for Biomedical Research, Medical University

of Vienna, Austria. **B Podesser**, K Trescher, O Bernecker, Ch Holzinger,

K Binder, I Schor, P Bergmann, H Kassal, E Dunkel (20)

26. Escorts Heart Institute, New Delhi, India. **N Trehan, Z Meharwal**, R Malhotra,

M Goel, B Ku-mer, S Bazaz, N Bake, A Singh, Y Mishka, R Gupta, S

Basumatary (19)

27. Silesian Centre for Heart Disease, Zabrze, Poland. **M Zembala**, B Szafron, J

Pacholewicz, M Krason, A Farmas, J Wojarski, B Zych, I Jaworska (10)

28. Szpital Wojewodzki 2, Rzeszow, Poland. **K Widenka**, I Szymanik, M Kolwca,

W Mazur, A Kurowicki, S Zurek, T Stacel, (6)

Table S1 Unit costs used to value resource use data collected from ART

| Resource category | Unit cost (2017/18 GBP/£) | Assumptions and Source |
| --- | --- | --- |
| Health care contact at follow-up |  |  |
| GP clinic attendance | 37.00 | Unit Costs of Health and Social Care 2018 (section 10.8b). Per surgery consultation lasting 9.22 minutes. |
| Visits to practice nurse | 10.85 | Unit Costs of Health and Social Care 2018 (section 1.3). Per patient contact lasting 15.5 minutes |
| Outpatient clinic attendance | 133.68 | NHS Reference Costs – Total Outpatient Attendance - Cardiology outpatient clinic (320). |
| Cardiac rehabilitation clinic attendance | 90.76 | NHS Reference Costs – Total Outpatient Attendance - Cardiac rehabilitation outpatient clinic (327). |
| Hospital re-admission bed day | 337.36 | NHS Reference Costs – Index - Non-elective inpatient excess bed day cost. Weighted average of all admission types. |
| Medications at follow-up (per day) |  |  |
| Aspirin | 0.01 | 75mg per day for duration of follow-up. |
| Clopidogrel | 0.02 | 75 mg per day for a period of three months unless otherwise stated. British National Formulary. |
| GpIIb/IIIa agent | 1.95 | Assumed drug is Ticagrelor (Brilique) at 180mg per day for duration of follow-up. |
| Warfarin | 0.03 | 6mg per day for duration of follow-up. |
| Beta-blockers | 0.01 | Assumed drug is Biprosolol at 5mg per day for duration of follow-up. |
| Calcium-channel antagonists | 0.01 | Assumed drug is Amlodipine at 5mg per day for duration of follow-up. |
| Nitrates | 0.06 | Assumed drug is Isosorbide Mononitrate at 40mg per day for duration of follow-up. |
| Potassium channel activators | 0.10 | Assumed drug is Nicorandil at 40mg per day for duration of follow-up. |
| Statins | 0.03 | Assumed half of patients received Simvastatin at 40mg per day and half Atorvastatin at 80mg per day for duration of follow-up. |
| Other lipid lowering drugs | 0.20 | Assumed drug is Ezetimibe at 10mg per day -up. |
| ACE inhibitors | 0.01 | Assumed drug is Ramipril at 5mg per day. |
| Angiotensin-II antagonists | 0.04 | Assumed half of patients received Losartan at 100mg per day and half Candesartan at 32mg per day for duration of follow-up. |
| Diuretics | 0.03 | Assumed drug is Furosemide at 40mg per day for a period of four weeks after surgery. |
| Digoxin | 0.03 | 250mcg per day for duration of follow-up. |
| Amiodarone | 0.11 | 600mg per day for duration of follow-up. |
| Adverse events at follow-up requiring admission | |  |
| Myocardial infarction | 2265.21 | NHS Reference Costs – Non-elective inpatient - Actual or Suspected Myocardial Infarction (weighted average across CC scores) (EB10). |
| Cerebrovascular accident | 3652.18 | NHS Reference Costs – Non-elective inpatient - Cerebrovascular Accident, Nervous System Infections or Encephalopathy (weighted average across CC scores) (AA22). |
| Further CABG | 10518.95 | NHS Reference Costs – Non-elective inpatient – Complex / Major / Standard Coronary Artery Bypass Graft (weighted average across CC scores) (ED26/7/8). |
| Further PCI | 3834.62 | NHS Reference Costs – Non-elective inpatient – Complex / Standard Percutaneous Transluminal Coronary Angioplasty (weighted average across CC scores) (EY40/1). |
| Revascularisation with catheterisation only | 3742.41 | NHS Reference Costs – Non-elective inpatient - Complex / Standard Cardiac Catheterisation Angioplasty (weighted average across CC scores) (EY42/3). |
| Major bleed | 3218.57 | Assumed to require surgery for investigation and to incur the same cost as sternal wound infection requiring reconstruction. |
| Other adverse events | 337.36 per day | Costed by associated length of hospital stay using the cost of a non-elective inpatient excess bed day. |
| Adverse events at follow-up without admission | |  |
| Myocardial infarction | 1239.63 | NHS Reference Costs - Day case - Actual or Suspected Myocardial Infarction, General day case (weighted average across CC scores) (EB10). |
| Cerebrovascular accident | 468.29 | NHS Reference Costs – Day case - Cerebrovascular Accident, Nervous System Infections or Encephalopathy (weighted average across CC scores) (AA22). |
| Further PCI | 2129.76 | NHS Reference Costs– Day case - Complex / Standard Percutaneous Transluminal Coronary Angioplasty (weighted average across CC scores) (EY40/1). |
| Major bleed | 3218.57 | Assumed to require surgery for investigation and to incur the same cost as sternal wound infection requiring reconstruction. |
| Revascularisation with catheterisation only | 1086.17 | NHS Reference Costs– Day case - Complex / Standard Cardiac Catheterisation (weighted average across CC scores) (EY42/3). |
| Emergency investigations | 288.84 | NHS Reference Costs– A&E category 4 or 5 treatment (weighted average). Used as an estimate for day hospital cost for patients not admitted. |
| Theatre Resource Use |  |  |
| Operating theatre time (per minute) | 13.35 | Average of theatre running costs (inclusive of staff) divided by theatre hours for cardiology at 8 boards in Scotland carrying out CABG (cardiac surgery). |
| Drugs for anaesthetic induction | 4.86 | Assuming 1mg Fentanyl , 5mg Midazolam, 1 amp (4mg) Pancuronium bromide |
| Muscle relaxant maintenance | 9.00 | Assuming 2 amps of Pancuronium bromide. |
| Inhalational anaesthetics (per minute) | 0.00 | Assuming Isoflurane at a concentration of 50% and fresh gas flow of 3 litres per min. |
| Consumables (all procedures) | 426.10 | Gray et al. 2017 inflated to 2017/18 prices using the GDP deflator index. |
| Additional consumables (on-pump procedures) | 106.53 | Gray et al. 2017 inflated to 2017/18 prices using the GDP deflator index. |
| Bypass machine time (per hour) | 15.57 | Machine purchase cost plus maintenance contract annuitised assuming a machine lifespan of 7 years and assuming a 3.5% discount rate. Annual usage of machine is assumed to be eight hours per day, five days per week. |
| Unit of red blood cells | 122.42 | Assuming 294ml mean volume per unit. |
| Unit of platelets | 197.05 | Assuming 297ml mean volume per unit. |
| Unit of fresh frozen plasma | 29.03 | Assuming 246ml mean volume per unit. |
| Unit of cryoprecipitate (pooled) | 181.15 | Assuming 187ml mean volume per unit |
| Laboratory issuing costs per blood product unit | 2.13 | Campbell et al. 2015 inflated to 2017/18 prices using the GDP deflator index. |
| Cell saver machine (per hour) | 1.47 | Machine purchase cost plus maintenance contract annuitised assuming a machine lifespan of 7 years and assuming a 3.5% discount rate. Annual usage of machine is assumed to be eight hours per day, five days per week. |
| Cell saver consumables | 106.53 | Gray et al. 2017 inflated to 2017/18 prices using the GDP deflator index. |
| Aprotinin (per dose) | 341.27 | Assuming one-off cost for 6 million Kallikrein Inhibitor Units intravenously; 2012/13 cost inflated to 2017/18 prices using the GDP deflator index. |
| Post-theatre Resource Use |  |  |
| Ventilator | 38.25 | Dasta et al. 2005 inflated to 2017/18 prices using the GDP deflator index. |
| Intra-aortic balloon pump plus pressure transducer (per insertion) | 596.54 | Gray et al. 2017 inflated to 2017/18 prices using the GDP deflator index. |
| *Inotrope drug days* |  |  |
| Adrenaline | 16.46 | Assuming average dose of 0.08 mcg/kg/min, 4mg in total volume 50ml with 5% Dextrose |
| Noradrenaline | 2.01 | Assuming average dose of 0.05 mcg/kg/min, 4mg in total volume 50ml with 5% Dextrose. |
| Dobutamine | 8.90 | Assuming average dose of 6 mcg/kg/min, 250mg in total volume 50ml with 5% |
| Renal support therapy (per day) | 271.06 | NHS reference cost – Renal - Haemodialysis for Acute Kidney Injury, 19 years and over (LE01A) |
| *Haemofiltration (single dose)* |  |  |
| Mannitol | 6.27 | Assumes a one off dose of 0.2-2g/kg intravenously. |
| Frusemide | 0.21 | Assumes a one off dose of 20-50mg intravenously. |
| *Treatment of sternal wound infection* |  |  |
| Antibiotics for superficial SWI (per course) | 7.80 | Assuming antibacterial protocol of 1g Flucloxacillin IV 4 times daily for 2 days; and either 2g Flucloxacillin IV once daily, 500mg Cefalexin 3 times daily, or 450mg Clindamycin 3 times daily for 5 days. |
| Antibiotics for deep sternal wound infection (per course) | 105.93 | Assuming antibacterial protocol of 500mg Meropenem and 600mg iv for 3 doses, then 600mg Teicoplanin IV once daily, then 3 times daily for 7 days. |
| VAC Dressing (per episode) | 310.63 | Gray et al. 2017 inflated to 2017/18 prices using the GDP deflator index. Includes VAC canister, dressing and machine and assumes three applications. Costs provided by trial participating hospital. |
| Debridement procedures | 1616.93 | Assumed to require two hours of theatre time and prophylactic antibiotics. Time costed using theatre and anaesthetic drug costs |
| Surgical reconstruction | 3218.57 | Assumed to require four hours of theatre time and prophylactic antibiotics. Time costed using theatre and anaesthetic drug costs |
| SAEs during index admission |  |  |
| Myocardial infarction | 880.40 | NHS Reference Costs – Non-elective inpatient - Actual or Suspected Myocardial Infarction (weighted average across CC scores) (EB10), less inpatient bed days costed using average excess bed day cost for the same currency code (weighted average across CC scores). |
| Cerebrovascular accident | 1031.93 | NHS Reference Costs – Non-elective inpatient - Cerebrovascular Accident, Nervous System Infections or Encephalopathy (weighted average across CC scores) (AA22), less inpatient bed days costed using average excess bed day cost for the same currency codes (weighted average across CC scores). |
| Further CABG | 8250.59 | NHS Reference Costs – Non-elective inpatient – Complex / Major / Standard Coronary Artery Bypass Graft (weighted average across CC scores) (ED26/7/8), less inpatient bed days costed using average excess bed day cost for the same currency codes (weighted average across CC scores). |
| Further PCI | 2483.04 | NHS Reference Costs – Non-elective inpatient – Complex / Standard Percutaneous Transluminal Coronary Angioplasty (weighted average across CC scores) (EY40/1), less inpatient bed days costed using average excess bed day cost for the same currency codes (weighted average across CC scores). |
| Cardiac catheterisation only | 1528.35 | NHS Reference Costs – Non-elective inpatient - Complex / Standard Cardiac Catheterisation Angioplasty (weighted average across CC scores) (EY42/3), less inpatient bed days costed using average excess bed day cost for the same currency codes (weighted average across CC scores). |
| Major bleed | 3218.57 | Assumed to require surgery for investigation and to incur the same cost as sternal wound infection requiring reconstruction. |
| Inpatient bed days |  |  |
| Intensive Therapy Unit | 693.12 | NHS Reference costs - Critical care - weighted average across all unventilated critical care service codes (CCU01-91) (Currency code: XC07Z). |
| High Dependency Unit | 394.15 | Gray et al. 2017 inflated to 2017/18 prices using the GDP deflator index. |
| Cardiac Ward | 321.71 | NHS Reference costs - Non Elective Inpatients Excess Bed Days - Complex / Major / Standard Coronary Artery Bypass Graft (weighted average across CC scores) (ED26/7/8) |
| Post-hospital discharge |  |  |
| Other hospital (per day) | 321.71 | Assumed same bed day cost as for cardiac ward above. |
| Nursing home (per day) | 157.86 | Unit Costs of Health and Social Care 2018 (section 1.3). |
| Rehabilitation unit (total cost) | 2991.24 | Unit Costs of Health and Social Care 2014 (section 1.6) inflated to 2017/18 prices using the GDP deflator index. Assumes an average stay of 33 days. |

Table S2 EQ-5D-3L responses (n (%)) by dimension for available cases up to 10 years follow-up

|  | Mobility | | Self-care | | Usual activity | | Pain/discomfort | | Anxiety / depression | |
| --- | --- | --- | --- | --- | --- | --- | --- | --- | --- | --- |
| Baseline | SITA | BITA | SITA | BITA | SITA | BITA | SITA | BITA | SITA | BITA |
| 1 | 853 (56.4) | 811 (54.2) | 1358 (90.1) | 1363 (91.4) | 731 (48.5) | 709 (47.8) | 513 (34.0) | 475 (32.1) | 828 (55.1) | 815 (54.8) |
| 2 | 646 (42.7) | 679 (45.4) | **139** (9.2) | 125 (8.4) | 667 (44.3) | 649 (43.7) | 911 (60.5) | 927 (62.7) | 620 (41.3) | 607 (40.8) |
| 3 | 13 (0.9) | 5 (0.3) | 11 (0.7) | 4 (0.3) | 109 (7.2) | 126 (8.5) | 83 (5.5) | 77 (5.2) | 55 (3.7) | 64 (4.3) |
| p | 0.069 | | 0.140 | | 0.441 | | 0.458 | | 0.662 | |
| Year 1 |  |  |  |  |  |  |  |  |  |  |
| 1 | 1103 (76.9) | 1083 (77.0) | 1355 (94.5) | 1320 (94.2) | 1098 (76.7) | 1080 (77.2) | 958 (67.1) | 964 (68.9) | 1106 (77.7) | 1089 (78.1) |
| 2 | 329 (22.9) | 322 (22.9) | 76 (5.3) | 79 (5.6) | 312 (21.8) | 290 (20.7) | 444 (31.1) | 418 (29.9) | 295 (20.7) | 285 (20.4) |
| 3 | 2 (0.1) | 1 (0.1) | 3 (0.2) | 3 (0.2) | 21 (1.5) | 29 (2.1) | 26 (1.8) | 18 (1.3) | 22 (1.5) | 20 (1.4) |
| p | 0.854 | | 0.925 | | 0.392 | | 0.371 | | 0.951 | |
| Year 2 |  |  |  |  |  |  |  |  |  |  |
| 1 | 1073 (76.8) | 1047 (77.0) | 1330 (94.9) | 1283 (94.6) | 1093 (78.2) | 1075 (79.3) | 1013 (72.7) | 964 (71.2) | 1111 (79.7) | 1086 (80.1) |
| 2 | 324 (23.2) | 312 (22.9) | 69 (4.9) | 69 (5.1) | 280 (20.0) | 254 (18.7) | 356 (25.5) | 365 (27.0) | 268 (19.2) | 252 (18.6) |
| 3 | 1 (0.1) | 1 (0.1) | 2 (0.1) | 4 (0.3) | 25 (1.8) | 26 (1.9) | 25 (1.8) | 24 (1.8) | 15 (1.1) | 18 (1.3) |
| p | 0.989 | | 0.678 | | 0.683 | | 0.692 | | 0.769 | |
| Year 3 |  |  |  |  |  |  |  |  |  |  |
| 1 | 1011 (76.1) | 981 (75.7) | 1234 (93.1) | 1208 (93.6) | 1032 (77.8) | 994 (77.0) | 961 (72.6) | 912 (70.6) | 1073 (81.2) | 1029 (80.1) |
| 2 | 314 (23.6) | 313 (24.2) | 89 (6.7) | 79 (6.1) | 271 (20.4) | 274 (21.2) | 341 (25.8) | 359 (27.8) | 234 (17.7) | 238 (18.5) |
| 3 | 3 (0.2) | 2 (0.2) | 2 (0.2) | 4 (0.3) | 23 (1.7) | 23 (1.8) | 22 (1.7) | 20 (1.5) | 15 (1.1) | 17 (1.3) |
| p | 0.877 | | 0.578 | | 0.878 | | 0.491 | | 0.769 | |
| Year 4 |  |  |  |  |  |  |  |  |  |  |
| 1 | 874 (73.6) | 850 (72.2) | 1094 (92.2) | 1086 (92.3) | 887 (75.0) | 876 (74.7) | 813 (69.0) | 807 (69.1) | 942 (80.2) | 912 (78.4) |
| 2 | 311 (26.2) | 325 (27.6) | 86 (7.3) | 82 (7.0) | 272 (23.0) | 264 (22.5) | 335 (28.4) | 340 (29.1) | 216 (18.4) | 228 (19.6) |
| 3 | 3 (0.3) | 2 (0.2) | 6 (0.5) | 8 (0.7) | 23 (1.9) | 33 (2.8) | 30 (2.5) | 21 (1.8) | 16 (1.4) | 24 (2.1) |
| p | 0.673 | | 0.832 | | 0.379 | | 0.448 | | 0.306 | |
| Year 5 |  |  |  |  |  |  |  |  |  |  |
| 1 | 732 (65.7) | 749 (67.1) | 989 (88.7) | 1014 (90.9) | 763 (68.6) | 769 (69.0) | 686 (61.6) | 688 (61.8) | 856 (77.5) | 815 (73.8) |
| 2 | 380 (34.1) | 365 (32.7) | 120 (10.8) | 95 (8.5) | 311 (28.0) | 318 (28.5) | 384 (34.5) | 393 (35.3) | 226 (20.5) | 269 (24.3) |
| 3 | 2 (0.2) | 2 (0.2) | 6 (0.5) | 6 (0.5) | 38 (3.4) | 27 (2.4) | 43 (3.9) | 33 (3.0) | 23 (2.1) | 21 (1.9) |
| p | 0.781 | | 0.200 | | 0.375 | | 0.491 | | 0.089 | |
| Year 6 |  |  |  |  |  |  |  |  |  |  |
| 1 | 720 (68.3) | 708 (68.0) | 943 (89.5) | 931 (89.6) | 767 (72.7) | 735 (70.9) | 692 (65.8) | 666 (64.2) | 816 (77.3) | 785 (75.9) |
| 2 | 331 (31.4) | 329 (31.6) | 109 (10.3) | 98 (9.4) | 257 (24.4) | 277 (26.7) | 332 (31.6) | 338 (32.6) | 225 (21.3) | 229 (22.1) |
| 3 | 3 (0.3) | 4 (0.4) | 2 (0.2) | 10 (1.0) | 31 (2.9) | 25 (2.4) | 28 (2.7) | 33 (3.2) | 14 (1.3) | 20 (1.9) |
| p | 0.919 | | 0.053 | | 0.383 | | 0.653 | | 0.476 | |
| Year 7 |  |  |  |  |  |  |  |  |  |  |
| 1 | 589 (65.4) | 594 (66.7) | 814 (90.3) | 792 (89.4) | 645 (71.3) | 616 (69.3) | 599 (66.9) | 566 (64.0) | 692 (77.1) | 643 (72.7) |
| 2 | 309 (34.3) | 294 (33.0) | 84 (9.3) | 88 (9.9) | 234 (25.9) | 247 (27.8) | 272 (30.4) | 299 (33.8) | 199 (22.2) | 230 (26.0) |
| 3 | 3 (0.3) | 3 (0.3) | 3 (0.3) | 6 (0.7) | 26 (2.9) | 26 (2.9) | 24 (2.7) | 19 (2.1) | 7 (0.8) | 11 (1.2) |
| p | 0.844 | | 0.53 | | 0.645 | | 0.256 | | 0.090 | |
| Year 8 |  |  |  |  |  |  |  |  |  |  |
| 1 | 546 (66.3) | 571 (67.3) | 736 (89.4) | 763 (90.3) | 586 (71.4) | 582 (68.6) | 541 (66.0) | 552 (65.5) | 632 (77.0) | 647 (76.8) |
| 2 | 276 (33.5) | 275 (32.4) | 83 (10.1) | 76 (9.0) | 210 (25.6) | 235 (27.7) | 263 (32.1) | 262 (31.1) | 177 (21.6) | 183 (21.7) |
| 3 | 2 (0.2) | 3 (0.4) | 4 (0.5) | 6 (0.7) | 25 (3.0) | 31 (3.7) | 16 (2.0) | 29 (3.4) | 12 (1.5) | 12 (1.4) |
| p | 0.824 | | 0.636 | | 0.444 | | 0.169 | | 0.995 | |
| Year 9 |  |  |  |  |  |  |  |  |  |  |
| 1 | 477 (65.0) | 507 (67.2) | 643 (88.1) | 677 (90.6) | 498 (67.9) | 520 (69.3) | 466 (63.6) | 494 (65.3) | 556 (76.5) | 570 (75.9) |
| 2 | 254 (34.6) | 243 (32.2) | 83 (11.4) | 66 (8.8) | 214 (29.2) | 207 (27.6) | 241 (32.9) | 250 (33.1) | 164 (22.6) | 175 (23.3) |
| 3 | 3 (0.4) | 4 (0.5) | 4 (0.5) | 4 (0.5) | 21 (2.9) | 23 (3.1) | 26 (3.5) | 12 (1.6) | 7 (1.0) | 6 (0.8) |
| p | 0.597 | | 0.27 | | 0.784 | | 0.055 | | 0.897 | |
| Year 10 |  |  |  |  |  |  |  |  |  |  |
| 1 | 497 (60.8) | 526 (63.6) | 679 (83.2) | 710 (86.2) | 529 (64.7) | 543 (65.9) | 471 (57.7) | 518 (63.1) | 586 (72.2) | 620 (75.5) |
| 2 | 311 (38.1) | 297 (35.9) | 122 (15.0) | 104 (12.6) | 254 (31.1) | 248 (30.1) | 310 (38.0) | 271 (33.0) | 212 (26.1) | 191 (23.3) |
| 3 | 9 (1.1) | 4 (0.5) | 15 (1.8) | 10 (1.2) | 34 (4.2) | 33 (4.0) | 35 (4.3) | 32 (3.9) | 14 (1.7) | 10 (1.2) |
| p | 0.222 | | 0.214 | | 0.887 | | 0.083 | | 0.263 | |

*Table S3 Mean undiscounted EQ-5Q-3L index by year of follow-up*

|  | SITA | BITA | BITA vs SITA*  Mean difference (95% CI, p value) |
| --- | --- | --- | --- |
| Baseline | 0.73 | 0.72 | -0.01 (-0, 0; 0.410) |
| Year 1 | 0.85 | 0.85 | -0.00 (-0, 0; 0.580) |
| Year 2 | 0.86 | 0.85 | -0.01 (-0, 0; 0.148) |
| Year 3 | 0.84 | 0.84 | 0.00 (-0, 0; 0.941) |
| Year 4 | 0.81 | 0.81 | -0.00 (-0, 0; 0.694) |
| Year 5 | 0.77 | 0.77 | 0.01 (-0, 0; 0.481) |
| Year 6 | 0.77 | 0.76 | -0.01 (-0, 0; 0.427) |
| Year 7 | 0.76 | 0.74 | -0.03 (-0, -0; 0.049) |
| Year 8 | 0.73 | 0.73 | -0.00 (-0, 0; 0.973) |
| Year 9 | 0.69 | 0.72 | 0.03 (-0, 0; 0.080) |
| Year 10 | 0.66 | 0.66 | 0.00 (-0, 0; 0.828) |

**Estimated differences are adjusted for baseline EQ-5D-3L index*

Table S4 Version 2 of the 36-Item Short Survey (SF-36v2) by year

|  | Baseline | | | Year 5 | | | Year 10 | | |
| --- | --- | --- | --- | --- | --- | --- | --- | --- | --- |
|  | SITA | BITA | BITA vs SITA | SITA | BITA | BITA vs SITA* | SITA | BITA | BITA vs SITA* |
| Physical functioning | 56 | 57 | 1 (-1, 2; 0.475) | 69 | 67 | -2 (-5, 2; 0.292) | 59 | 62 | 3 (-1, 8; 0.153) |
| Role-physical | 47 | 47 | 1 (-1, 2; 0.235) | 68 | 68 | -1 (-5, 3; 0.607) | 59 | 60 | 0 (-4, 5; 0.941) |
| Bodily Pain | 65 | 65 | 0 (-1, 2; 0.855) | 78 | 77 | -1 (-3, 1; 0.377) | 73 | 73 | 1 (-4, 5; 0.659) |
| General Health | 58 | 58 | 0 (-2, 2; 0.990) | 60 | 59 | -0 (-4, 3; 0.785) | 54 | 55 | 2 (-3, 6; 0.432) |
| Vitality | 49 | 48 | -2 (-3, -0; 0.032) | 57 | 56 | 0 (-3, 3; 0.830) | 51 | 53 | 2 (-2, 7; 0.327) |
| Social Functioning | 63 | 63 | -0 (-2, 2; 0.735) | 78 | 77 | -1 (-4, 2; 0.491) | 70 | 70 | 0 (-5, 5; 0.962) |
| Role - Emotional | 65 | 66 | 1 (-1, 3; 0.495) | 78 | 77 | -1 (-4, 3; 0.772) | 69 | 70 | 0 (-6, 6; 0.982) |
| Mental Health | 66 | 66 | -0 (-1, 1; 0.905) | 74 | 73 | -1 (-3, 2; 0.570) | 68 | 67 | -1 (-5, 3; 0.512) |

**Estimated differences are adjusted for baseline SF-36v2.*

Table S5 Shortened World Health Organisation Rose Angina Questionnaire by year

|  | Has pain or discomfort in chest | | | Pain when walking ordinary pace on the level* | | | Pain when walking uphill or hurrying* | | |
| --- | --- | --- | --- | --- | --- | --- | --- | --- | --- |
|  | SITA | BITA | BITA vs SITA** | SITA | BITA | BITA vs SITA** | SITA | BITA | BITA vs SITA** |
| Baseline | 80% | 79% | -0.01 (-0.03, 0.02; 0.693) | 45% | 44% | 0.45 (0.43, 0.48; 0.000) | 87% | 89% | 0.02 (-0.01, 0.05; 0.229) |
| Year 1 | 31% | 35% | 0.03 (-0.00, 0.07; 0.070) | 10% | 12% | 0.02 (-0.02, 0.07; 0.285) | 41% | 41% | 0.00 (-0.07, 0.08; 0.896) |
| Year 2 | 29% | 32% | 0.02 (-0.02, 0.06; 0.248) | 16% | 16% | -0.01 (-0.08, 0.06; 0.831) | 44% | 51% | 0.07 (-0.02, 0.15; 0.107) |
| Year 3 | 30% | 31% | 0.01 (-0.03, 0.06; 0.547) | 19% | 18% | -0.00 (-0.06, 0.06; 0.879) | 44% | 48% | 0.04 (-0.05, 0.13; 0.374) |
| Year 4 | 29% | 30% | 0.01 (-0.04, 0.06; 0.739) | 19% | 20% | 0.02 (-0.05, 0.08; 0.586) | 55% | 53% | -0.01 (-0.11, 0.08; 0.769) |
| Year 5 | 33% | 35% | 0.02 (-0.04, 0.09; 0.487) | 20% | 21% | 0.01 (-0.06, 0.07; 0.809) | 55% | 53% | -0.02 (-0.11, 0.07; 0.626) |
| Year 6 | 30% | 33% | 0.03 (-0.03, 0.09; 0.318) | 20% | 21% | 0.01 (-0.06, 0.08; 0.854) | 50% | 58% | 0.08 (-0.03, 0.19; 0.136) |
| Year 7 | 28% | 34% | 0.06 (0.00, 0.12; 0.037) | 22% | 20% | -0.02 (-0.12, 0.07; 0.598) | 48% | 55% | 0.06 (-0.06, 0.19; 0.285) |
| Year 8 | 31% | 34% | 0.02 (-0.06, 0.10; 0.583) | 19% | 24% | 0.05 (-0.05, 0.15; 0.281) | 48% | 57% | 0.09 (-0.06, 0.24; 0.210) |
| Year 9 | 32% | 26% | -0.05 (-0.19, 0.08; 0.428) | 25% | 17% | -0.08 (-0.27, 0.10; 0.336) | 53% | 56% | 0.03 (-0.19, 0.25; 0.760) |
| Year 10 | 32% | 31% | -0.01 (-0.09, 0.08; 0.825) | 21% | 18% | -0.02 (-0.11, 0.07; 0.646) | 53% | 60% | - 1. -0.08, 0.22; 0.314) |

**Responses reported only for patients answering, “Yes” to question one. **Estimated differences are adjusted for baseline responses.*

Table S6 Cumulative cost-effectiveness by year

|  | Mean total cost | | | Mean QALYs | | |  |
| --- | --- | --- | --- | --- | --- | --- | --- |
|  | SITA | BITA | BITA vs SITA | SITA | BITA | BITA vs SITA* | ICER |
| Baseline-year 1 | 11391 | 12378 | 987 (388, 1586; 0.002) | 0.77 | 0.76 | -0.000 (-0.01, 0.01; 0.938) | -1199862 |
| Year 1-2 | 12169 | 13117 | 947 (268, 1627; 0.008) | 1.58 | 1.57 | -0.004 (-0.03, 0.02; 0.762) | -181829 |
| Year 2-3 | 12805 | 13798 | 993 (280, 1706; 0.008) | 2.35 | 2.34 | -0.010 (-0.05, 0.03; 0.646) | -89984 |
| Year 3-4 | 13437 | 14435 | 998 (265, 1732; 0.010) | 3.08 | 3.07 | -0.011 (-0.07, 0.05; 0.715) | -84207 |
| Year 4-5 | 14089 | 15044 | 955 (161, 1749; 0.020) | 3.76 | 3.74 | -0.008 (-0.08, 0.07; 0.824) | -89226 |
| Year 5-6 | 14573 | 15706 | 1133 (242, 2025; 0.015) | 4.39 | 4.37 | -0.007 (-0.10, 0.09; 0.879) | -102965 |
| Year 6-7 | 15139 | 16305 | 1166 (285, 2047; 0.012) | 4.99 | 4.96 | -0.016 (-0.12, 0.09; 0.757) | -55032 |
| Year 7-8 | 15697 | 16794 | 1096 (150, 2043; 0.025) | 5.56 | 5.52 | -0.025 (-0.15, 0.10; 0.684) | -36376 |
| Year 8-9 | 16142 | 17277 | 1135 (220, 2050; 0.017) | 6.08 | 6.06 | -0.014 (-0.15, 0.13; 0.843) | -52459 |
| Year 9-10 | 16587 | 17705 | 1118 (195, 2041; 0.020) | 6.56 | 6.55 | -0.002 (-0.16, 0.15; 0.978) | -97422 |

**Estimated differences in QALYs are adjusted for baseline EQ-5D-3L index*


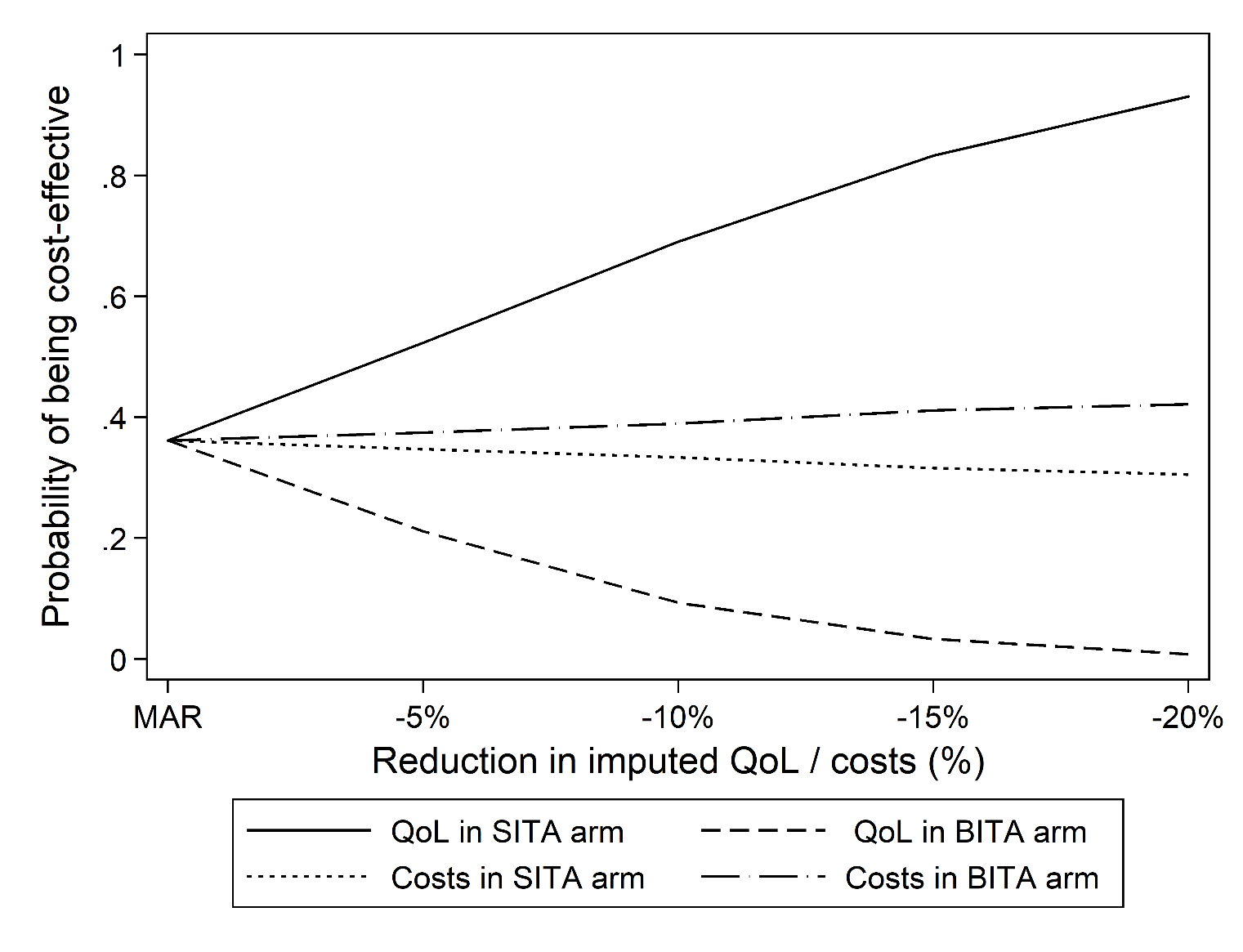


Figure S1 Probability of BITA being cost-effective at a willingness to pay of £20,000 per QALY by MNAR sensitivity parameters

Table S7 Resource use, costs, quality adjusted life years and cost-effectiveness at 10 year follow-up (“Per protocol” Analysis)

|  | Mean resource use / n of adverse events at year 10 | | | Mean total cost at year 10 | | |
| --- | --- | --- | --- | --- | --- | --- |
|  | SITA  (n =1494) | BITA  (n=1294) | BITA vs SITA  Mean difference (95% CI, p value) | SITA  (n =1494) | BITA  (n=1294) | BITA vs SITA  Mean difference (95% CI, p value) |
| Initial surgery |  |  |  |  |  |  |
| Index admission |  |  |  | 8806 | 9539 | 732 (104, 1361; 0.024) |
| Discharge cost |  |  |  | 570 | 524 | -46 (-344, 252; 0.754) |
| Healthcare contacts |  |  |  |  |  |  |
| GP visits | 32 | 31 | -1.09 (-3, 1; 0.247) | 1416 | 1377 | -39 (-140, 63; 0.440) |
| Nurse visits | 14 | 14 | 0.08 (-2, 2; 0.932) | 175 | 175 | -0 (-17, 17; 0.994) |
| Outpatient clinic visits | 10 | 11 | 0.98 (-0, 2; 0.190) | 1604 | 1775 | 171 (30, 312; 0.020) |
| Cardiac rehabilitation visits | 10 | 10 | -0.81 (-4, 2; 0.569) | 988 | 912 | -76 (-403, 251; 0.636) |
| Number of nights in hospital* | 2 | 2 | 0.47 (-0, 1; 0.147) | 657 | 823 | 166 (-97, 429; 0.205) |
| All health care contacts |  |  |  | 4840 | 5062 | 222 (-162, 607; 0.245) |
| Medications |  |  |  |  |  |  |
| Total medication | 36 | 36 | 0.20 (-1, 1; 0.758) | 217 | 222 | 5 (-7, 17; 0.387) |
| SAE treatment** |  |  |  |  |  |  |
| Myocardial infarction | 49 | 49 | 1.15 (0.8, 1.7; 0.477) | 72 | 75 | 4 (-33, 40; 0.842) |
| Cerebrovascular accident | 63 | 38 | 0.70 (0.5, 1.0; 0.078) | 135 | 95 | -40 (-99, 20; 0.180) |
| Further CABG | 0 | 2 |  | 0 | 16 | 16 (-4, 37; 0.118) |
| Further PCI | 141 | 114 | 0.93 (0.7, 1.2; 0.585) | 304 | 293 | -10 (-95, 75; 0.804) |
| Cardiac catheterisation | 66 | 47 | 0.82 (0.6, 1.2; 0.305) | 102 | 101 | -1 (-50, 48; 0.956) |
| Sternal wound problems | 37 | 59 | 1.84 (1.2, 2.8; 0.004) | 88 | 306 | 217 (66, 369; 0.007) |
| Major bleed | 10 | 10 | 1.15 (0.5, 2.8; 0.748) | 49 | 83 | 35 (-46, 116; 0.387) |
| Other AEs (cost of hospital stay only) | 1429 | 1451 | 1.17 (1.1, 1.3; 0.000) | 2150 | 2504 | 354 (-211, 919; 0.209) |
| Death (cost of hospital stay only) | 304 | 238 | 0.90 (0.8, 1.1; 0.243) | 400 | 367 | -33 (-276, 210; 0.785) |
| All adverse event costs |  |  |  | 3299 | 3841 | 542 (-148, 1232; 0.118) |
| All costs |  |  |  | 17732 | 19188 | 1456 (315, 2597; 0.015) |
|  |  |  |  |  |  |  |
| Discounted total cost |  |  |  | 16547 | 17934 | 1387 (336, 2438; 0.012) |
|  |  |  |  |  |  |  |
| Life years |  |  |  | 9.03 | 9.09 | 0.05 (-0.15, 0.25; 0.586) |
| QALYs*** |  |  |  | 6.60 | 6.62 | 0.03 (-0.1, 0.2; 0.682) |
|  |  |  |  |  |  |  |
| ICER |  |  |  | 41421 | | |
| Probability of cost-effectiveness |  |  |  | 41% | | |

*Number of nights in hospital exclusive of those associated with an "other" adverse event or death. **SAE treatment is that occurring in the follow-up period only. The cost of SAEs which occurred during the index admission is included in the cost of index admission. ***Estimated differences in QALYs are adjusted for baseline EQ-5D-3L index


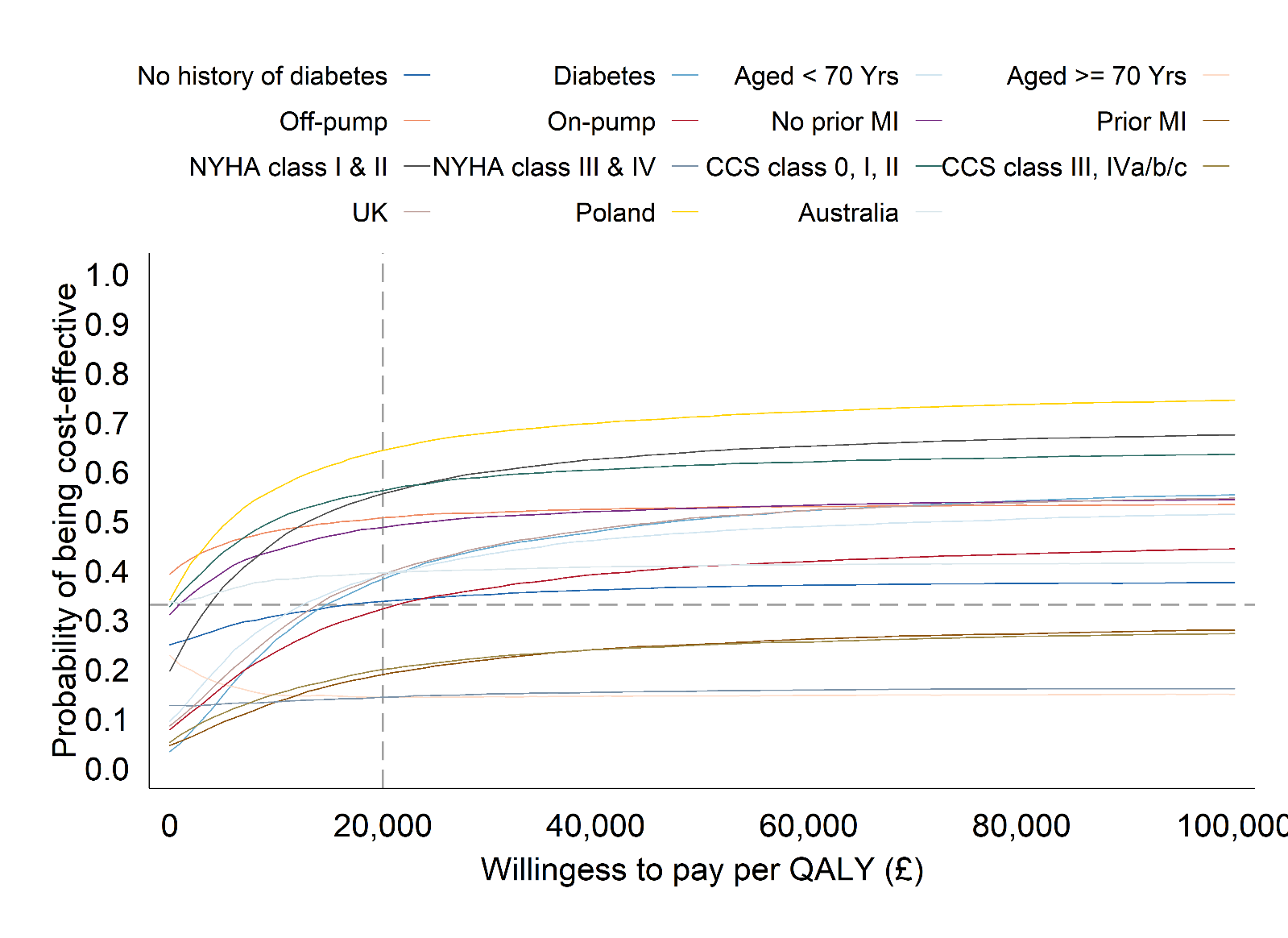


Figure S2 Cost-effectiveness acceptability curves by patient subgroup

Table S8 Cost-effectiveness by subgroups: diabetes

|  | No history of diabetes | | | Diabetes | | |
| --- | --- | --- | --- | --- | --- | --- |
|  | SITA | BITA | BITA vs SITA | SITA | BITA | BITA vs SITA |
| Initial surgery |  |  |  |  |  |  |
| Index admission | 8708 | 9311 | 603 (-71, 1277; 0.077) | 9181 | 9995 | 815 (7, 1623; 0.048) |
| Discharge cost | 626 | 533 | -92 (-463, 278; 0.612) | 353 | 527 | 174 (-176, 523; 0.315) |
| Healthcare contacts |  |  |  |  |  |  |
| GP visits | 1383 | 1354 | -28 (-116, 59; 0.509) | 1561 | 1566 | 6 (-148, 159; 0.937) |
| Nurse visits | 163 | 170 | 7 (-13, 28; 0.481) | 208 | 193 | -15 (-57, 28; 0.489) |
| Outpatient clinic visits | 1554 | 1654 | 99 (-92, 291; 0.295) | 1739 | 2048 | 309 (-83, 701; 0.117) |
| Cardiac rehabilitation visits | 1043 | 955 | -88 (-381, 206; 0.543) | 725 | 789 | 64 (-694, 823; 0.862) |
| Number of nights in hospital* | 601 | 681 | 79 (-265, 424; 0.639) | 858 | 1212 | 354 (-244, 952; 0.233) |
| All health care contacts | 4744 | 4814 | 70 (-401, 540; 0.762) | 5090 | 5809 | 719 (-123, 1560; 0.090) |
| Medications |  |  |  |  |  |  |
| Total medication | 219 | 221 | 2 (-12, 16; 0.762) | 213 | 228 | 14 (-7, 35; 0.176) |
| SAE treatment** |  |  |  |  |  |  |
| Myocardial infarction | 76 | 62 | -14 (-58, 29; 0.502) | 66 | 83 | 17 (-38, 73; 0.523) |
| Cerebrovascular accident | 133 | 92 | -41 (-102, 21; 0.187) | 117 | 71 | -46 (-137, 46; 0.314) |
| Further CABG | 0 | 18 | 18 (-5, 41; 0.126) | 0 | 0 | . |
| Further PCI | 291 | 290 | -1 (-94, 92; 0.985) | 338 | 333 | -5 (-111, 101; 0.924) |
| Cardiac catheterisation | 107 | 98 | -9 (-75, 57; 0.784) | 77 | 136 | 59 (-17, 135; 0.124) |
| Sternal wound problems | 65 | 148 | 83 (-17, 183; 0.101) | 219 | 683 | 464 (-48, 975; 0.074) |
| Major bleed | 39 | 86 | 47 (-44, 138; 0.301) | 79 | 20 | -59 (-182, 64; 0.331) |
| Other AEs (cost of hospital stay only) | 2119 | 2086 | -33 (-631, 565; 0.910) | 2465 | 3299 | 833 (-227, 1894; 0.118) |
| Death (cost of hospital stay only) | 384 | 338 | -46 (-316, 224; 0.729) | 480 | 448 | -32 (-298, 234; 0.806) |
| All adverse event costs | 3213 | 3217 | 4 (-730, 738; 0.991) | 3841 | 5073 | 1231 (107, 2356; 0.033) |
| All costs | 17510 | 18096 | 586 (-603, 1775; 0.320) | 18678 | 21631 | 2953 (1058, 4848; 0.004) |
|  |  |  |  |  |  |  |
| Discounted total costs | 16345 | 16930 | 584 (-498, 1667; 0.277) | 17378 | 20164 | 2786 (1072, 4499; 0.003) |
|  |  |  |  |  |  |  |
| Life years | 9.10 | 9.11 | 0.00 (-0.18, 0.19; 0.958) | 8.78 | 8.87 | 0.08 (-0.38, 0.55; 0.716) |
| QALYs*** | 6.67 | 6.61 | -0.03 (-0.2, 0.1; 0.688) | 6.23 | 6.32 | 0.06 (-0.3, 0.5; 0.760) |
|  |  | | |  | | |
| ICER | -18340 | | | 45642 | | |
| Probability of cost-effectiveness | 33% | | | 39% | | |

**Number of nights in hospital exclusive of those associated with an "other" adverse event or death. **SAE treatment is that occurring in the follow-up period only. The cost of SAEs which occurred during the index admission is included in the cost of index admission. ***Estimated differences in QALYs are adjusted for baseline EQ-5D-3L index*

Table S9 Cost-effectiveness by subgroups: age-groups

|  | Aged < 70 Yrs | | | Aged >= 70 Yrs | | |
| --- | --- | --- | --- | --- | --- | --- |
|  | SITA | BITA | BITA vs SITA | SITA | BITA | BITA vs SITA |
| Initial surgery |  |  |  |  |  |  |
| Index admission | 8612 | 9157 | 545 (52, 1037; 0.032) | 9366 | 10371 | 1005 (-66, 2077; 0.064) |
| Discharge cost | 359 | 415 | 56 (-81, 193; 0.410) | 1098 | 859 | -239 (-1348, 870; 0.659) |
| Healthcare contacts |  |  |  |  |  |  |
| GP visits | 1412 | 1456 | 44 (-25, 113; 0.200) | 1456 | 1262 | -194 (-415, 26; 0.081) |
| Nurse visits | 154 | 162 | 7 (-14, 28; 0.477) | 224 | 215 | -9 (-78, 59; 0.785) |
| Outpatient clinic visits | 1615 | 1752 | 138 (-73, 348; 0.189) | 1552 | 1736 | 184 (-208, 577; 0.339) |
| Cardiac rehabilitation visits | 909 | 835 | -74 (-571, 423; 0.761) | 1127 | 1142 | 16 (-495, 526; 0.950) |
| Number of nights in hospital* | 565 | 605 | 40 (-164, 244; 0.689) | 916 | 1379 | 463 (-220, 1147; 0.173) |
| All health care contacts | 4655 | 4810 | 155 (-306, 616; 0.496) | 5275 | 5735 | 460 (-300, 1220; 0.222) |
| Medications |  |  |  |  |  |  |
| Total medication | 215 | 230 | 15 (0, 29; 0.048) | 223 | 201 | -23 (-40, -5; 0.012) |
| SAE treatment** |  |  |  |  |  |  |
| Myocardial infarction | 71 | 70 | -0 (-27, 26; 0.970) | 83 | 59 | -24 (-90, 42; 0.463) |
| Cerebrovascular accident | 105 | 62 | -43 (-100, 14; 0.132) | 194 | 159 | -35 (-166, 95; 0.580) |
| Further CABG | 0 | 18 | 18 (-6, 43; 0.134) | 0 | 0 | . |
| Further PCI | 349 | 322 | -26 (-146, 93; 0.654) | 178 | 238 | 60 (-50, 171; 0.271) |
| Cardiac catheterisation | 109 | 121 | 11 (-49, 71; 0.706) | 74 | 69 | -6 (-82, 71; 0.881) |
| Sternal wound problems | 105 | 287 | 182 (-10, 373; 0.062) | 89 | 245 | 156 (-23, 335; 0.084) |
| Major bleed | 43 | 30 | -12 (-68, 43; 0.648) | 63 | 181 | 118 (-119, 356; 0.313) |
| Other AEs (cost of hospital stay only) | 1714 | 2099 | 385 (-243, 1013; 0.219) | 3491 | 3160 | -331 (-1093, 430; 0.376) |
| Death (cost of hospital stay only) | 152 | 198 | 46 (-79, 171; 0.457) | 1074 | 829 | -246 (-889, 398; 0.436) |
| All adverse event costs | 2648 | 3208 | 560 (-147, 1268; 0.115) | 5246 | 4939 | -307 (-1518, 903; 0.603) |
| All costs | 16490 | 17820 | 1330 (499, 2162; 0.003) | 21208 | 22104 | 896 (-1413, 3205; 0.428) |
|  |  |  |  |  |  |  |
| Discounted total costs | 15404 | 16679 | 1275 (544, 2006; 0.001) | 19719 | 20591 | 872 (-1339, 3084; 0.421) |
|  |  |  |  |  |  |  |
| Life years | 9.29 | 9.35 | 0.06 (-0.13, 0.25; 0.515) | 8.33 | 8.2 | -0.13 (-0.43, 0.16; 0.366) |
| QALYs*** | 6.76 | 6.78 | 0.02 (-0.2, 0.2; 0.836) | 6.06 | 5.89 | -0.13 (-0.4, 0.2; 0.365) |
|  |  | | |  | | |
| ICER | 72855 | | | -6465 | | |
| Probability of cost-effectiveness | 39% | | | 15% | | |

**Number of nights in hospital exclusive of those associated with an "other" adverse event or death. **SAE treatment is that occurring in the follow-up period only. The cost of SAEs which occurred during the index admission is included in the cost of index admission. ***Estimated differences in QALYs are adjusted for baseline EQ-5D-3L index*

Table S10 Cost-effectiveness by subgroups: on/off pump

|  | Off-pump | | | On-pump | | |
| --- | --- | --- | --- | --- | --- | --- |
|  | SITA | BITA | BITA vs SITA | SITA | BITA | BITA vs SITA |
| Initial surgery |  |  |  |  |  |  |
| Index admission | 8888 | 8912 | 24 (-783, 831; 0.951) | 8836 | 10032 | 1196 (692, 1700; 0.000) |
| Discharge cost | 622 | 526 | -96 (-329, 137; 0.400) | 525 | 538 | 13 (-501, 528; 0.958) |
| Healthcare contacts |  |  |  |  |  |  |
| GP visits | 1208 | 1208 | -0 (-65, 65; 1.000) | 1573 | 1547 | -26 (-147, 94; 0.654) |
| Nurse visits | 199 | 195 | -4 (-23, 16; 0.712) | 157 | 162 | 5 (-21, 30; 0.696) |
| Outpatient clinic visits | 1952 | 2067 | 115 (-115, 344; 0.307) | 1362 | 1523 | 161 (-35, 356; 0.102) |
| Cardiac rehabilitation visits | 1436 | 934 | -503 (-847, -158; 0.007) | 661 | 910 | 249 (-136, 634; 0.194) |
| Number of nights in hospital* | 556 | 892 | 336 (-193, 865; 0.200) | 733 | 753 | 20 (-177, 216; 0.838) |
| All health care contacts | 5351 | 5296 | -55 (-511, 400; 0.801) | 4486 | 4894 | 408 (-168, 983; 0.156) |
| Medications |  |  |  |  |  |  |
| Total medication | 224 | 230 | 6 (-6, 18; 0.297) | 213 | 218 | 4 (-14, 22; 0.623) |
| SAE treatment** |  |  |  |  |  |  |
| Myocardial infarction | 94 | 78 | -16 (-65, 34; 0.517) | 61 | 60 | -1 (-27, 26; 0.944) |
| Cerebrovascular accident | 155 | 101 | -55 (-133, 24; 0.160) | 113 | 79 | -34 (-98, 31; 0.290) |
| Further CABG | 0 | 16 | 16 (-8, 41; 0.180) | 0 | 12 | 12 (-13, 37; 0.335) |
| Further PCI | 344 | 318 | -26 (-157, 104; 0.678) | 276 | 293 | 17 (-66, 99; 0.678) |
| Cardiac catheterisation | 113 | 134 | 20 (-57, 98; 0.595) | 92 | 90 | -2 (-67, 63; 0.956) |
| Sternal wound problems | 43 | 265 | 222 (29, 416; 0.027) | 140 | 289 | 148 (-42, 339; 0.121) |
| Major bleed | 63 | 70 | 7 (-97, 111; 0.893) | 38 | 71 | 32 (-75, 140; 0.540) |
| Other AEs (cost of hospital stay only) | 2248 | 2730 | 482 (-128, 1092; 0.115) | 2189 | 2163 | -25 (-884, 834; 0.952) |
| Death (cost of hospital stay only) | 605 | 472 | -133 (-478, 213; 0.433) | 275 | 287 | 12 (-273, 297; 0.931) |
| All adverse event costs | 3666 | 4184 | 518 (-225, 1261; 0.161) | 3185 | 3344 | 160 (-785, 1104; 0.730) |
| All costs | 18751 | 19148 | 397 (-738, 1532; 0.473) | 17245 | 19026 | 1781 (180, 3382; 0.031) |
|  |  |  |  |  |  |  |
| Discounted total costs | 17419 | 17754 | 335 (-716, 1385; 0.512) | 16132 | 17889 | 1757 (323, 3190; 0.019) |
|  |  |  |  |  |  |  |
| Life years | 9.01 | 9.04 | 0.03 (-0.25, 0.32; 0.805) | 9.05 | 9.06 | 0.01 (-0.22, 0.24; 0.923) |
| QALYs*** | 6.51 | 6.52 | 0.03 (-0.2, 0.3; 0.805) | 6.65 | 6.64 | -0.00 (-0.2, 0.2; 0.986) |
|  |  | | |  | | |
| ICER | 12183 | | | -991946 | | |
| Probability of cost-effectiveness | 51% | | | 32% | | |

**Number of nights in hospital exclusive of those associated with an "other" adverse event or death. **SAE treatment is that occurring in the follow-up period only. The cost of SAEs which occurred during the index admission is included in the cost of index admission. ***Estimated differences in QALYs are adjusted for baseline EQ-5D-3L index*

Table S11 Cost-effectiveness by subgroups: baseline history of myocardial infarction

|  | No prior MI | | | Prior MI | | |
| --- | --- | --- | --- | --- | --- | --- |
|  | SITA | BITA | BITA vs SITA | SITA | BITA | BITA vs SITA |
| Initial surgery |  |  |  |  |  |  |
| Index admission | 8631 | 9200 | 569 (-25, 1163; 0.059) | 9059 | 9864 | 805 (169, 1441; 0.015) |
| Discharge cost | 506 | 582 | 76 (-189, 341; 0.560) | 635 | 458 | -177 (-765, 411; 0.541) |
| Healthcare contacts |  |  |  |  |  |  |
| GP visits | 1422 | 1412 | -10 (-118, 98; 0.853) | 1427 | 1394 | -33 (-118, 52; 0.427) |
| Nurse visits | 175 | 174 | -1 (-20, 19; 0.940) | 171 | 178 | 6 (-17, 30; 0.588) |
| Outpatient clinic visits | 1584 | 1649 | 65 (-128, 259; 0.490) | 1616 | 1897 | 282 (6, 557; 0.045) |
| Cardiac rehabilitation visits | 1095 | 1072 | -23 (-454, 408; 0.913) | 807 | 682 | -125 (-416, 165; 0.383) |
| Number of nights in hospital* | 726 | 704 | -21 (-310, 268; 0.881) | 580 | 965 | 385 (-151, 921; 0.151) |
| All health care contacts | 5002 | 5013 | 11 (-559, 580; 0.970) | 4602 | 5116 | 515 (67, 962; 0.026) |
| Medications |  |  |  |  |  |  |
| Total medication | 220 | 217 | -3 (-21, 14; 0.697) | 214 | 231 | 17 (-5, 38; 0.119) |
| SAE treatment** |  |  |  |  |  |  |
| Myocardial infarction | 86 | 50 | -36 (-75, 4; 0.078) | 59 | 93 | 34 (-11, 78; 0.129) |
| Cerebrovascular accident | 128 | 84 | -44 (-115, 26; 0.209) | 131 | 92 | -38 (-110, 33; 0.279) |
| Further CABG | 0 | 11 | 11 (-13, 35; 0.338) | 0 | 17 | 17 (-16, 50; 0.296) |
| Further PCI | 355 | 294 | -60 (-175, 54; 0.289) | 235 | 310 | 75 (-88, 238; 0.352) |
| Cardiac catheterisation | 127 | 92 | -35 (-93, 24; 0.230) | 65 | 129 | 64 (-10, 138; 0.086) |
| Sternal wound problems | 131 | 226 | 95 (-54, 244; 0.201) | 62 | 351 | 289 (67, 511; 0.013) |
| Major bleed | 49 | 81 | 32 (-72, 136; 0.529) | 47 | 53 | 6 (-81, 92; 0.893) |
| Other AEs (cost of hospital stay only) | 2126 | 2043 | -83 (-739, 573; 0.796) | 2301 | 2843 | 542 (-226, 1310; 0.158) |
| Death (cost of hospital stay only) | 422 | 343 | -79 (-455, 298; 0.671) | 380 | 394 | 15 (-211, 240; 0.896) |
| All adverse event costs | 3424 | 3226 | -198 (-876, 480; 0.553) | 3279 | 4282 | 1003 (6, 2000; 0.049) |
| All costs | 17783 | 18237 | 454 (-829, 1737; 0.472) | 17789 | 19952 | 2162 (669, 3655; 0.006) |
|  |  |  |  |  |  |  |
| Discounted total costs | 16550 | 17039 | 489 (-675, 1654; 0.395) | 16639 | 18655 | 2016 (632, 3399; 0.006) |
|  |  |  |  |  |  |  |
| Life years | 9.12 | 9.18 | 0.06 (-0.15, 0.27; 0.568) | 8.91 | 8.86 | -0.05 (-0.28, 0.17; 0.632) |
| QALYs*** | 6.65 | 6.65 | 0.03 (-0.2, 0.2; 0.794) | 6.46 | 6.38 | -0.08 (-0.3, 0.1; 0.430) |
|  |  | | |  | | |
| ICER | 19027 | | | -25443 | | |
| Probability of cost-effectiveness | 49% | | | 19% | | |

**Number of nights in hospital exclusive of those associated with an "other" adverse event or death. **SAE treatment is that occurring in the follow-up period only. The cost of SAEs which occurred during the index admission is included in the cost of index admission. ***Estimated differences in QALYs are adjusted for baseline EQ-5D-3L index*

Table S12 Cost-effectiveness by subgroups: NYHA class

|  | NYHA class I & II | | | NYHA class III & IV | | |
| --- | --- | --- | --- | --- | --- | --- |
|  | SITA | BITA | BITA vs SITA | SITA | BITA | BITA vs SITA |
| Initial surgery |  |  |  |  |  |  |
| Index admission | 8869 | 9513 | 643 (104, 1183; 0.021) | 8631 | 9344 | 713 (-242, 1667; 0.135) |
| Discharge cost | 584 | 450 | -135 (-370, 100; 0.248) | 480 | 817 | 338 (-580, 1255; 0.454) |
| Healthcare contacts |  |  |  |  |  |  |
| GP visits | 1478 | 1479 | 2 (-88, 92; 0.966) | 1217 | 1145 | -72 (-243, 99; 0.390) |
| Nurse visits | 165 | 164 | -1 (-20, 19; 0.933) | 206 | 216 | 11 (-35, 56; 0.628) |
| Outpatient clinic visits | 1556 | 1609 | 53 (-108, 213; 0.503) | 1742 | 2235 | 493 (-30, 1016; 0.064) |
| Cardiac rehabilitation visits | 957 | 949 | -8 (-354, 339; 0.965) | 1014 | 797 | -217 (-651, 216; 0.310) |
| Number of nights in hospital* | 700 | 739 | 39 (-255, 332; 0.788) | 517 | 1050 | 533 (-39, 1105; 0.066) |
| All health care contacts | 4855 | 4940 | 85 (-364, 534; 0.699) | 4697 | 5444 | 747 (108, 1385; 0.024) |
| Medications |  |  |  |  |  |  |
| Total medication | 215 | 224 | 8 (-5, 22; 0.228) | 225 | 219 | -6 (-28, 15; 0.538) |
| SAE treatment** |  |  |  |  |  |  |
| Myocardial infarction | 70 | 55 | -15 (-48, 19; 0.380) | 91 | 110 | 19 (-39, 77; 0.506) |
| Cerebrovascular accident | 136 | 97 | -39 (-96, 17; 0.166) | 104 | 54 | -50 (-138, 38; 0.251) |
| Further CABG | 0 | 17 | 17 (-6, 41; 0.134) | 0 | 0 | . |
| Further PCI | 294 | 309 | 15 (-73, 102; 0.734) | 320 | 270 | -49 (-163, 65; 0.382) |
| Cardiac catheterisation | 80 | 90 | 10 (-34, 54; 0.645) | 169 | 166 | -3 (-143, 138; 0.970) |
| Sternal wound problems | 107 | 279 | 172 (-20, 364; 0.077) | 77 | 265 | 187 (-80, 455; 0.161) |
| Major bleed | 36 | 49 | 13 (-70, 96; 0.751) | 93 | 141 | 48 (-136, 232; 0.594) |
| Other AEs (cost of hospital stay only) | 2060 | 2236 | 175 (-346, 697; 0.495) | 2744 | 2872 | 128 (-1502, 1758; 0.873) |
| Death (cost of hospital stay only) | 450 | 312 | -138 (-473, 197; 0.404) | 237 | 544 | 307 (41, 574; 0.026) |
| All adverse event costs | 3234 | 3444 | 210 (-434, 854; 0.508) | 3835 | 4423 | 588 (-1247, 2423; 0.514) |
| All costs | 17758 | 18570 | 812 (-217, 1842; 0.117) | 17868 | 20246 | 2378 (-105, 4862; 0.060) |
|  |  |  |  |  |  |  |
| Discounted total costs | 16572 | 17357 | 785 (-133, 1703; 0.090) | 16633 | 18917 | 2284 (-37, 4604; 0.053) |
|  |  |  |  |  |  |  |
| Life years | 9.03 | 9.13 | 0.10 (-0.12, 0.31; 0.357) | 8.99 | 8.76 | -0.23 (-0.46, 0.00; 0.053) |
| QALYs*** | 6.64 | 6.70 | 0.07 (-0.1, 0.2; 0.338) | 6.29 | 6.01 | -0.29 (-0.6, -0.0; 0.044) |
|  |  | | |  | | |
| ICER | 11016 | | | -7876 | | |
| Probability of cost-effectiveness | 57% | | | 15% | | |

**Number of nights in hospital exclusive of those associated with an "other" adverse event or death. **SAE treatment is that occurring in the follow-up period only. The cost of SAEs which occurred during the index admission is included in the cost of index admission. ***Estimated differences in QALYs are adjusted for baseline EQ-5D-3L index*

Table S13 Cost-effectiveness by subgroups: CSS class

|  | CCS class 0, I, II | | | CCS class III, IV a/b/c | | |
| --- | --- | --- | --- | --- | --- | --- |
|  | SITA | BITA | BITA vs SITA | SITA | BITA | BITA vs SITA |
| Initial surgery |  |  |  |  |  |  |
| Index admission | 8867 | 9486 | 619 (101, 1137; 0.021) | 8708 | 9452 | 744 (-187, 1674; 0.112) |
| Discharge cost | 476 | 453 | -23 (-215, 168; 0.805) | 757 | 703 | -54 (-993, 885; 0.907) |
| Healthcare contacts |  |  |  |  |  |  |
| GP visits | 1432 | 1393 | -39 (-130, 53; 0.390) | 1407 | 1431 | 24 (-76, 124; 0.624) |
| Nurse visits | 175 | 166 | -8 (-30, 13; 0.434) | 171 | 196 | 25 (1, 49; 0.041) |
| Outpatient clinic visits | 1532 | 1507 | -24 (-200, 151; 0.777) | 1748 | 2274 | 527 (37, 1016; 0.036) |
| Cardiac rehabilitation visits | 1045 | 950 | -95 (-572, 382; 0.686) | 793 | 839 | 45 (-153, 243; 0.640) |
| Number of nights in hospital* | 704 | 645 | -59 (-269, 151; 0.567) | 563 | 1164 | 601 (-150, 1351; 0.112) |
| All health care contacts | 4887 | 4662 | -225 (-792, 342; 0.422) | 4682 | 5904 | 1222 (561, 1883; 0.001) |
| Medications |  |  |  |  |  |  |
| Total medication | 216 | 216 | 0 (-14, 15; 0.963) | 222 | 236 | 15 (-1, 31; 0.072) |
| SAE treatment** |  |  |  |  |  |  |
| Myocardial infarction | 83 | 57 | -26 (-59, 6; 0.107) | 53 | 89 | 37 (-2, 75; 0.062) |
| Cerebrovascular accident | 144 | 92 | -52 (-110, 6; 0.078) | 96 | 77 | -19 (-94, 56; 0.615) |
| Further CABG | 0 | 20 | 20 (-7, 46; 0.134) | 0 | 0 | . |
| Further PCI | 303 | 264 | -39 (-106, 28; 0.241) | 298 | 379 | 81 (-51, 212; 0.219) |
| Cardiac catheterisation | 81 | 84 | 3 (-55, 61; 0.918) | 142 | 156 | 15 (-92, 121; 0.779) |
| Sternal wound problems | 113 | 310 | 197 (-21, 415; 0.075) | 73 | 202 | 129 (-26, 284; 0.098) |
| Major bleed | 53 | 46 | -7 (-104, 89; 0.878) | 37 | 122 | 85 (-30, 200; 0.140) |
| Other AEs (cost of hospital stay only) | 2231 | 2120 | -111 (-715, 494; 0.709) | 2133 | 2939 | 806 (-348, 1959; 0.163) |
| Death (cost of hospital stay only) | 474 | 382 | -92 (-441, 257; 0.592) | 247 | 323 | 76 (-76, 229; 0.312) |
| All adverse event costs | 3484 | 3376 | -108 (-927, 711; 0.789) | 3078 | 4288 | 1210 (-92, 2512; 0.067) |
| All costs | 17930 | 18193 | 263 (-1051, 1577; 0.684) | 17447 | 20584 | 3136 (1097, 5176; 0.004) |
|  |  |  |  |  |  |  |
| Discounted total costs | 16698 | 17061 | 363 (-795, 1520; 0.524) | 16333 | 19113 | 2780 (873, 4687; 0.006) |
|  |  |  |  |  |  |  |
| Life years | 9.00 | 9.06 | 0.07 (-0.18, 0.32; 0.571) | 9.10 | 9.01 | -0.09 (-0.34, 0.17; 0.492) |
| QALYs*** | 6.64 | 6.67 | 0.05 (-0.1, 0.2; 0.592) | 6.40 | 6.27 | -0.13 (-0.4, 0.2; 0.353) |
|  |  | | |  | | |
| ICER | 7846 | | | -21033 | | |
| Probability of cost-effectiveness | 56% | | | 20% | | |

**Number of nights in hospital exclusive of those associated with an "other" adverse event or death. **SAE treatment is that occurring in the follow-up period only. The cost of SAEs which occurred during the index admission is included in the cost of index admission. ***Estimated differences in QALYs are adjusted for baseline EQ-5D-3L index*

Table S14 Cost-effectiveness by subgroups: by selected country

|  | UK | | | Poland | | | Australia | | |
| --- | --- | --- | --- | --- | --- | --- | --- | --- | --- |
|  | SITA | BITA | BITA vs SITA | SITA | BITA | BITA vs SITA | SITA | BITA | BITA vs SITA |
| Initial surgery |  |  |  |  |  |  |  |  |  |
| Index admission | 8725 | 9284 | 559 (-295, 1414; 0.179) | 8534 | 9261 | 727 (410, 1044; 0.004) | 10613 | 11154 | 541 (-834, 1915; 0.439) |
| Discharge cost | 518 | 463 | -55 (-577, 468; 0.824) | 844 | 849 | 5 (-194, 205; 0.946) | 215 | 186 | -29 (-242, 184; 0.790) |
| Healthcare contacts |  |  |  |  |  |  |  |  |  |
| GP visits | 1200 | 1225 | 25 (-34, 83; 0.369) | 2002 | 2050 | 49 (-214, 311; 0.617) | 2954 | 2543 | -411 (-829, 7; 0.054) |
| Nurse visits | 239 | 242 | 3 (-18, 25; 0.730) | 49 | 48 | -1 (-28, 26; 0.903) | 77 | 21 | -56 (-163, 50; 0.299) |
| Outpatient clinic visits | 1702 | 1893 | 191 (13, 369; 0.038) | 1613 | 1804 | 191 (-202, 583; 0.221) | 1160 | 1034 | -125 (-360, 109; 0.293) |
| Cardiac rehabilitation visits | 1329 | 1266 | -63 (-605, 479; 0.804) | 298 | 189 | -110 (-399, 180; 0.355) | 430 | 450 | 20 (-213, 254; 0.863) |
| Number of nights in hospital* | 562 | 833 | 271 (-121, 662; 0.158) | 545 | 504 | -41 (-449, 367; 0.780) | 1129 | 472 | -658 (-1492, 176; 0.121) |
| All health care contacts | 5032 | 5459 | 427 (-34, 888; 0.067) | 4507 | 4595 | 88 (-656, 831; 0.730) | 5750 | 4520 | -1230 (-2283, -177; 0.022) |
| Medications |  |  |  |  |  |  |  |  |  |
| Total medication | 227 | 235 | 8 (-7, 23; 0.281) | 186 | 194 | 8 (-25, 41; 0.504) | 203 | 195 | -8 (-38, 22; 0.594) |
| SAE treatment** |  |  |  |  |  |  |  |  |  |
| Myocardial infarction | 91 | 82 | -10 (-53, 34; 0.639) | 36 | 31 | -5 (-73, 63; 0.855) | 72 | 47 | -25 (-128, 78; 0.636) |
| Cerebrovascular accident | 128 | 102 | -27 (-103, 50; 0.463) | 106 | 39 | -67 (-210, 76; 0.278) | 159 | 151 | -8 (-243, 227; 0.946) |
| Further CABG | 0 | 20 | 20 (-6, 47; 0.122) | 0 | 0 | . | 0 | 0 | . |
| Further PCI | 328 | 313 | -15 (-130, 100; 0.781) | 211 | 267 | 56 (-34, 147; 0.168) | 242 | 220 | -23 (-325, 280; 0.883) |
| Cardiac catheterisation | 126 | 128 | 2 (-83, 86; 0.968) | 48 | 76 | 28 (-48, 104; 0.384) | 0 | 0 | . |
| Sternal wound problems | 125 | 281 | 156 (-11, 323; 0.065) | 58 | 175 | 117 (-237, 471; 0.427) | 58 | 838 | 780 (-80, 1639; 0.075) |
| Major bleed | 57 | 79 | 22 (-76, 119; 0.634) | 52 | 0 | -52 (-149, 45; 0.224) | 0 | 58 | 58 (-57, 173; 0.324) |
| Other AEs (cost of hospital stay only) | 2801 | 2915 | 114 (-692, 920; 0.764) | 983 | 1035 | 51 (-376, 479; 0.766) | 1978 | 2577 | 599 (-891, 2089; 0.429) |
| Death (cost of hospital stay only) | 535 | 367 | -168 (-387, 51; 0.121) | 67 | 140 | 74 (-390, 537; 0.695) | 475 | 222 | -253 (-662, 156; 0.224) |
| All adverse event costs | 4192 | 4287 | 95 (-816, 1006; 0.825) | 1561 | 1763 | 202 (-897, 1302; 0.651) | 2984 | 4112 | 1128 (-949, 3204; 0.285) |
| All costs | 18694 | 19728 | 1035 (-537, 2606; 0.177) | 15631 | 16662 | 1031 (-177, 2238; 0.077) | 19765 | 20166 | 401 (-2518, 3320; 0.787) |
| Discounted total costs | 17365 | 18384 | 1019 (-439, 2476; 0.154) | 14658 | 15664 | 1006 (-72, 2084; 0.061) | 18444 | 19027 | 584 (-2073, 3240; 0.665) |
| Life years | 8.94 | 9.06 | 0.11 (-0, 0; 0.227) | 9.10 | 9.30 | 0.20 (-0, 0; 0.119) | 9.23 | 8.86 | -0.36 (-1, 0; 0.269) |
| QALYs*** | 6.39 | 6.40 | 0.02 (-0, 0; 0.810) | 6.79 | 6.96 | 0.20 (-0, 0; 0.091) | 6.59 | 6.54 | -0.06 (-1, 1; 0.846) |
| ICER | 41041 | | | 4954 | | | -10047 | | |
| Probability of cost-effectiveness | 40% | | | 65% | | | 39% | | |

**Number of nights in hospital exclusive of those associated with an "other" adverse event or death. **SAE treatment is that occurring in the follow-up period only. The cost of SAEs which occurred during the index admission is included in the cost of index admission. ***Estimated differences in QALYs are adjusted for baseline EQ-5D-3L index*

*
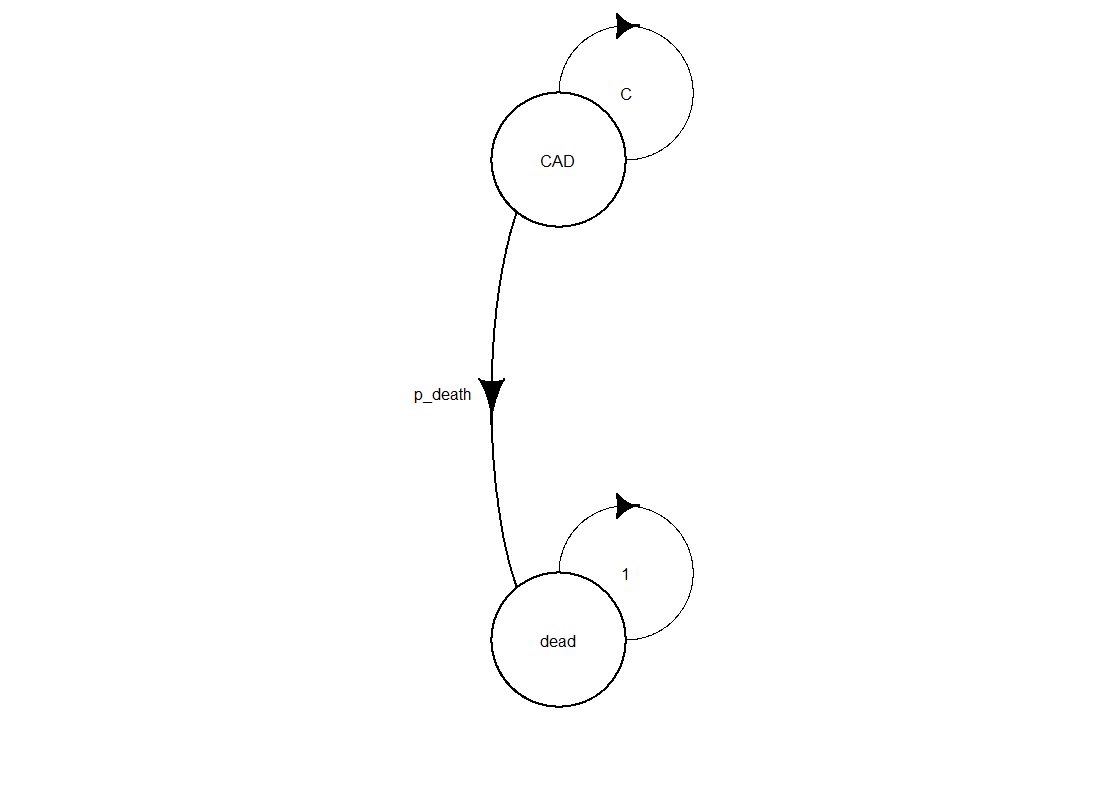
*

*Figure 3 Structure of the extrapolation model. CAD, coronary heart disease, p_death, is the probability of death at a given age estimated from extrapolated survival from the trial using Gompertz functions, C is equal to 1-p_death.*


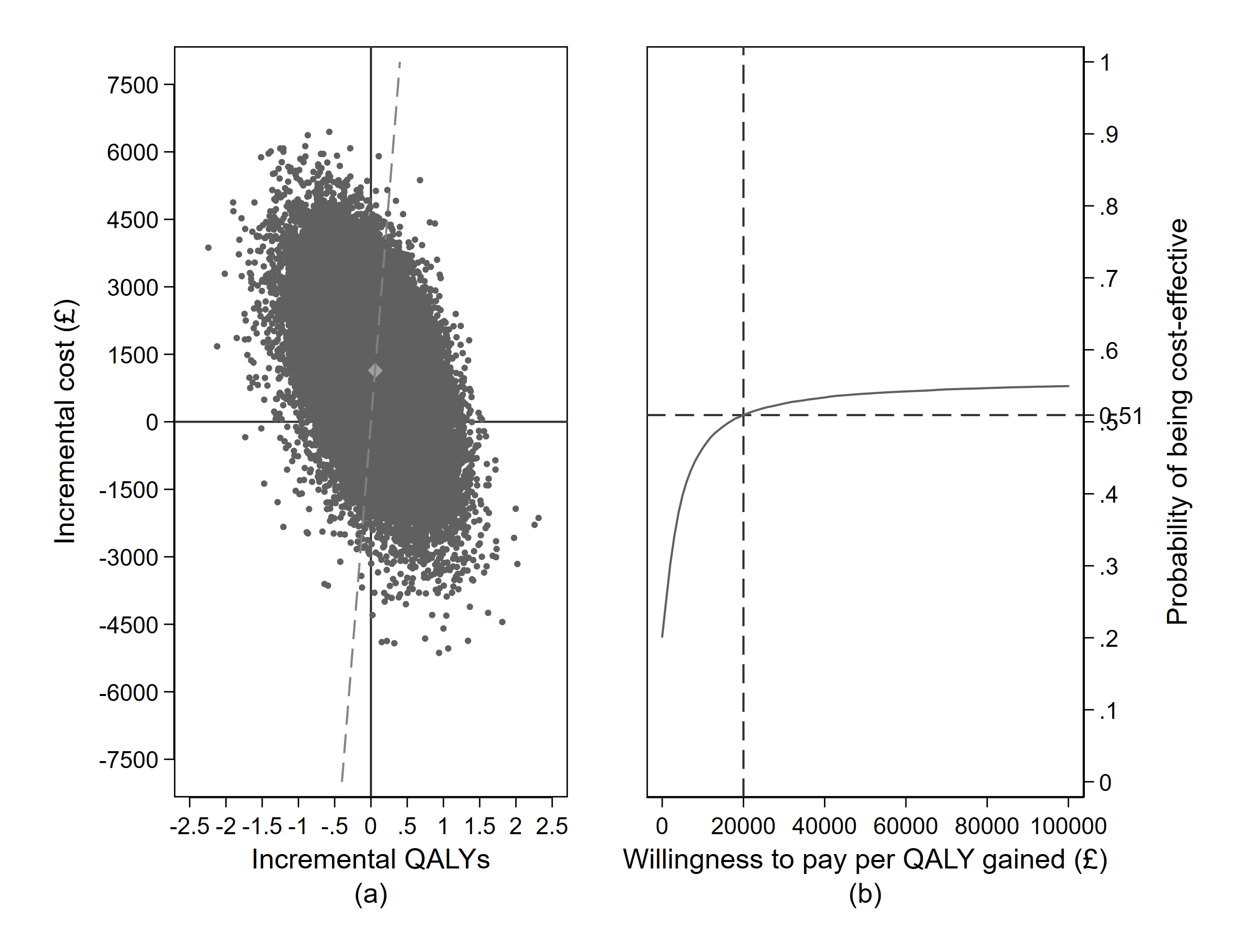


*Figure 4 a) Cost-effectiveness plane and b) cost-effectiveness acceptability curve from the extrapolation model’s probabilistic sensitivity analysis.*

*Caption: Figure 1a plots a series of simulations showing the likelihood that BITA yields more or fewer quality adjusted life years than SITA (X-axis), and costs more or less than SITA (Y-axis). Co-ordinates to the left and above the dashed line have a cost-effectiveness ratio above £20,000 per QALY gained; those to the right and below the line are less than £20,000 per QALY gained. Figure 1b shows the probability that BITA is cost-effective compared to SITA as the willingness to pay for each QALY gained is varied from £0 to £100,000, that is, as the dashed line is rotated around the origin.*
